# Supplementary material for: NF-κB subunits direct kinetically distinct transcriptional cascades in antigen receptor-activated B cells
Source: Nat Immunol. 2023 Jul 31;24(9):1552–64. doi: 10.1038/s41590-023-01561-7 (PMC10457194; doi:10.1038/s41590-023-01561-7)

nature immunology

Article https://doi.org/10.1038/s41590-023-01561-7

NF-κB subunits direct kinetically distinct transcriptional cascades in antigen receptor-activated B cells

In the format provided by the authors and unedited

Figure S1

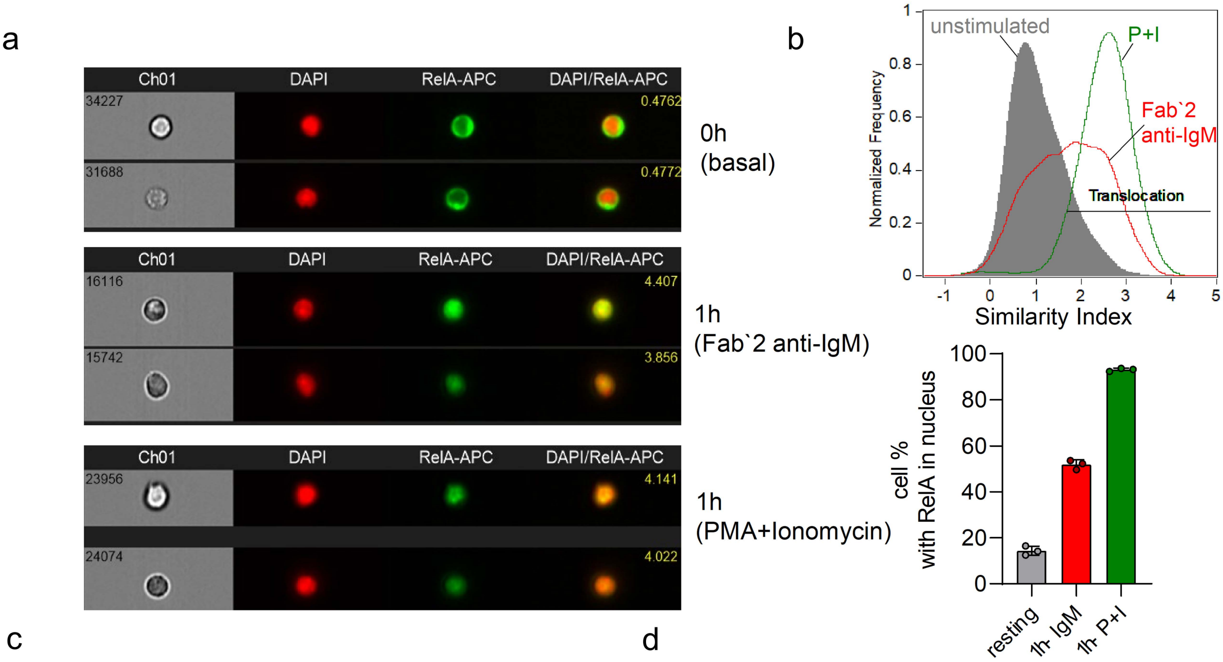

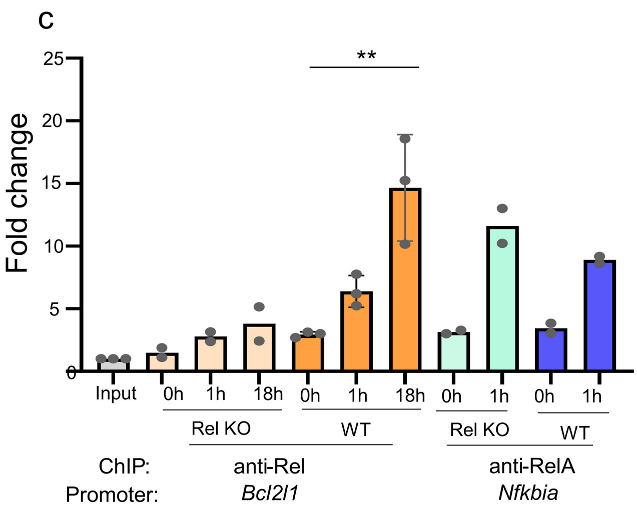

d

Library replicates	PFTags	Aligned tags	Non-redundant tags	Aligned tags %	Redundancy %
RelA 0h1	18006501	13726998	12973858	76.23	5.49
RelA 0h2	19297043	14975637	14319720	77.61	4.38
RelA 1h1	23244380	17836080	16854432	76.73	5.50
RelA 1h2	22444277	17489575	16917257	77.92	3.27
RelA 4h1	19378655	14804817	13021669	76.40	12.04
RelA 4h2	27177361	20942244	20054699	77.06	4.24
Rel 0h1	13014585	8422021	8034467	64.71	4.60
Rel 0h2	10775720	6948318	6661407	64.48	4.13
Rel 1h1	12243380	7734997	7428934	63.18	3.96
Rel 1h2	13532549	8679158	8180912	64.14	5.74
Rel 18h1	29183482	19220274	17578007	65.86	8.54
Rel 18h2	32455066	21305293	19926858	65.65	6.47

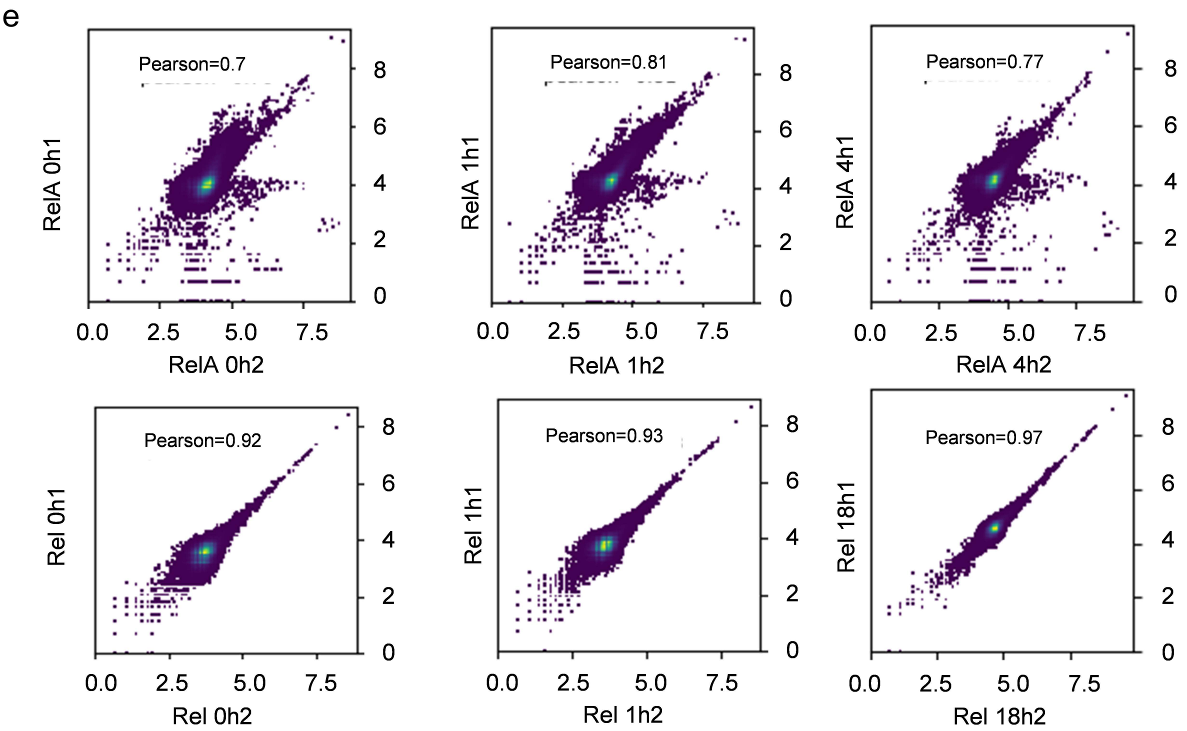

Figure S1

f

RelA 0h

Rank	Motif	P-value	log P-pvalue	% of Targets	% of Background	STD(Bg STD)	Best Match/Details
1	TTTCCCCG	1e-13	-3.137e+01	44.07%	21.84%	54.0bp (71.6bp)	RELB/MA1117.1/Jaspar(0.825) More Information | Similar Motifs Found
2	TAAAACGACAGC	1e-13	-3.107e+01	2.12%	0.00%	29.3bp (29.9bp)	RUNX-AML(Runt)/CD4+-PolII-ChIP-Seq(Barski_et_al.)/Homer(0.736) More Information | Similar Motifs Found
3	AAAATAGAACTG	1e-13	-3.042e+01	8.90%	0.99%	56.5bp (66.8bp)	SpiB(ETS)/OCILY3-SPIB-ChIP-Seq(GSE56857)/Homer(0.721) More Information | Similar Motifs Found
4	GGGGATTTCC	1e-12	-2.823e+01	6.36%	0.46%	63.9bp (63.2bp)	MF0003.1_REL_class/Jaspar(0.954) More Information | Similar Motifs Found

RelA 1h

Rank	Motif	P-value	log P-pvalue	% of Targets	% of Background	STD(Bg STD)	Best Match/Details
1	AAGCGGAAGT	1e-168	-3.870e+02	30.75%	13.42%	53.8bp (61.8bp)	PB0058.1_Sfpi1_1/Jaspar(0.951) More Information | Similar Motifs Found
2	GGGAATTCCCCT	1e-157	-3.626e+02	11.02%	2.19%	50.6bp (61.1bp)	NFkB-p65(RHD)/GM12787-p65-ChIP-Seq(GSE19485)/Homer(0.949) More Information | Similar Motifs Found
3	GAAGTGAAACTA	1e-68	-1.584e+02	2.80%	0.27%	51.4bp (65.1bp)	PU.1:IRF8(ETS:IRF)/pDC-Irf8-ChIP-Seq(GSE66899)/Homer(0.939) More Information | Similar Motifs Found
4	GTGGTTTCCC	1e-43	-1.004e+02	3.53%	0.80%	54.8bp (60.5bp)	RUNX1(Runt)/Jurkat-RUNX1-ChIP-Seq(GSE29180)/Homer(0.764) More Information | Similar Motifs Found
5	TCCCAGGGGA	1e-39	-9.017e+01	5.08%	1.68%	52.7bp (55.0bp)	EBF(EBF)/proBcell-EBF-ChIP-Seq(GSE21978)/Homer(0.897) More Information | Similar Motifs Found
6	TTATGCAAAT	1e-37	-8.722e+01	2.80%	0.58%	56.0bp (55.0bp)	Oct4(POU,Homeobox)/mES-Oct4-ChIP-Seq(GSE11431)/Homer(0.985) More Information | Similar Motifs Found
7	ATGAGTCATG	1e-37	-8.628e+01	11.15%	5.72%	55.0bp (58.7bp)	BATF(bZIP)/Th17-BATF-ChIP-Seq(GSE39756)/Homer(0.967) More Information | Similar Motifs Found
8	CGCTTCCTGT	1e-37	-8.534e+01	14.58%	8.31%	55.3bp (63.1bp)	ERG(ETS)/VCaP-ERG-ChIP-Seq(GSE14097)/Homer(0.787) More Information | Similar Motifs Found
9	CACGTGAC	1e-27	-6.445e+01	17.12%	11.10%	53.4bp (62.4bp)	USF1/MA0093.2/Jaspar(0.952) More Information | Similar Motifs Found
10	GTGCGAAA	1e-23	-5.354e+01	29.05%	22.08%	56.9bp (64.4bp)	Rbpj1(?)/Panc1-Rbpj1-ChIP-Seq(GSE47459)/Homer(0.782) More Information | Similar Motifs Found
11	CGCTATTT	1e-22	-5.222e+01	26.88%	20.20%	56.5bp (64.2bp)	Mef2c(MADS)/GM12878-Mef2c-ChIP-Seq(GSE32465)/Homer(0.754) More Information | Similar Motifs Found
12	TAACCACATCCT	1e-19	-4.557e+01	0.34%	0.01%	45.3bp (40.5bp)	ETS:RUNX(ETS,Runt)/Jurkat-RUNX1-ChIP-Seq(GSE17954)/Homer(0.881) More Information | Similar Motifs Found
13	AGAAAGCCCCTA	1e-17	-4.059e+01	3.74%	1.66%	56.5bp (57.9bp)	MF0003.1_REL_class/Jaspar(0.672) More Information | Similar Motifs Found
14	CAGTTCTGTTAT	1e-16	-3.829e+01	0.39%	0.02%	50.8bp (63.5bp)	PB0141.1_Isgf3g_2/Jaspar(0.710) More Information | Similar Motifs Found
15	TGACGTCA	1e-15	-3.593e+01	9.53%	6.13%	56.1bp (62.0bp)	CREB1/MA0018.3/Jaspar(0.947) More Information | Similar Motifs Found
16	CTATAATTAG	1e-15	-3.526e+01	2.04%	0.70%	52.8bp (59.6bp)	MEF2D/MA0773.1/Jaspar(0.885) More Information | Similar Motifs Found
17	ACATACGCAG	1e-14	-3.365e+01	32.56%	26.82%	55.1bp (61.3bp)	SCL(bHLH)/HPC7-Scl-ChIP-Seq(GSE13511)/Homer(0.530) More Information | Similar Motifs Found
18	ATGGAAACTT	1e-13	-3.137e+01	1.83%	0.64%	60.6bp (62.2bp)	NFAT(RHD)/Jurkat-NFATC1-ChIP-Seq(Jolma_et_al.)/Homer(0.832) More Information | Similar Motifs Found

RelA 4h

Rank	Motif	P-value	log P-pvalue	% of Targets	% of Background	STD(Bg STD)	Best Match/Details
1	GGGAATTCCCCA	1e-86	-1.993e+02	20.47%	4.81%	52.4bp (62.7bp)	NFkB-p65(RHD)/GM12787-p65-ChIP-Seq(GSE19485)/Homer(0.969) More Information | Similar Motifs Found
2	ACTTCCGCTT	1e-86	-1.991e+02	36.55%	14.24%	52.8bp (62.8bp)	PB0058.1_Sfpi1_1/Jaspar(0.949) More Information | Similar Motifs Found
3	GAAGTGAAAC	1e-43	-1.001e+02	6.98%	1.03%	50.8bp (65.0bp)	PU.1:IRF8(ETS:IRF)/pDC-Irf8-ChIP-Seq(GSE66899)/Homer(0.854) More Information | Similar Motifs Found
4	AAACCCCAGGGG	1e-30	-7.014e+01	14.04%	5.35%	51.9bp (60.7bp)	RUNX(Runt)/HPC7-Runx1-ChIP-Seq(GSE22178)/Homer(0.820) More Information | Similar Motifs Found
5	AGTCCCCTGGGA	1e-24	-5.645e+01	8.55%	2.69%	51.9bp (58.9bp)	EBF(EBF)/proBcell-EBF-ChIP-Seq(GSE21978)/Homer(0.941) More Information | Similar Motifs Found
6	CACGTGAC	1e-17	-3.922e+01	17.57%	9.77%	53.4bp (62.8bp)	USF1/MA0093.2/Jaspar(0.939) More Information | Similar Motifs Found
7	TTGAGTCA	1e-16	-3.734e+01	13.96%	7.23%	57.4bp (61.7bp)	Fosl2(bZIP)/3T3L1-Fosl2-ChIP-Seq(GSE56872)/Homer(0.928) More Information | Similar Motifs Found
8	TATGCAAATT	1e-15	-3.671e+01	3.53%	0.77%	55.4bp (61.6bp)	Oct11(POU,Homeobox)/NCIH1048-POU2F3-ChIP-seq(GSE115123)/Homer(0.965) More Information | Similar Motifs Found
9	ACCACAGCTC	1e-15	-3.649e+01	15.69%	8.58%	56.1bp (59.9bp)	RUNX-AML(Runt)/CD4+-PolII-ChIP-Seq(Barski_et_al.)/Homer(0.823) More Information | Similar Motifs Found
10	TTTCCAACCCAC	1e-13	-3.107e+01	3.69%	0.97%	55.7bp (61.1bp)	PB0167.1_Sox13_2/Jaspar(0.663) More Information | Similar Motifs Found
11	TCACACAGCC	1e-12	-2.935e+01	12.71%	6.95%	52.1bp (60.9bp)	POL009.1_DCE_S_II/Jaspar(0.675) More Information | Similar Motifs Found

g

Rel 0h

Rank	Motif	P-value	log P-pvalue	% of Targets	% of Background	STD(Bg STD)	Best Match/Details
1	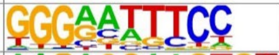	1e-36	-8.337e+01	20.47%	1.92%	45.7bp (65.6bp)	MF0003.1_REL_class/Jaspar(0.964) More Information | Similar Motifs Found
2	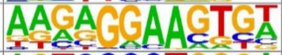	1e-22	-5.279e+01	14.17%	1.53%	55.6bp (65.6bp)	SpiB(ETS)/OCILY3-SPIB-ChIP-Seq(GSE56857)/Homer(0.929) More Information | Similar Motifs Found
3	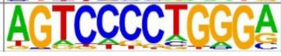	1e-13	-3.162e+01	7.48%	0.66%	50.5bp (57.6bp)	EBF(EBF)/proBcell-EBF-ChIP-Seq(GSE21978)/Homer(0.837) More Information | Similar Motifs Found

Rel 1h

Rank	Motif	P-value	log P-pvalue	% of Targets	% of Background	STD(Bg STD)	Best Match/Details
1	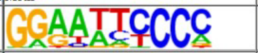	1e-166	-3.842e+02	37.24%	6.82%	49.8bp (62.7bp)	NFkB-p65(RHD)/GM12787-p65-ChIP-Seq(GSE19485)/Homer(0.951) More Information | Similar Motifs Found
2	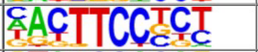	1e-79	-1.829e+02	39.76%	14.95%	52.6bp (63.1bp)	PB0058.1_Sfpi1_1/Jaspar(0.947) More Information | Similar Motifs Found
3	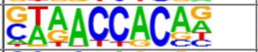	1e-30	-7.118e+01	11.71%	3.27%	59.3bp (63.6bp)	RUNX(Runt)/HPC7-Runx1-ChIP-Seq(GSE22178)/Homer(0.963) More Information | Similar Motifs Found
4	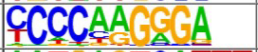	1e-25	-5.962e+01	17.76%	7.45%	56.2bp (62.5bp)	EBF2(EBF)/BrownAdipose-EBF2-ChIP-Seq(GSE97114)/Homer(0.906) More Information | Similar Motifs Found
5	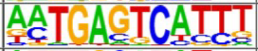	1e-23	-5.474e+01	7.27%	1.67%	53.7bp (60.8bp)	JUN(var.2)/MA0489.1/Jaspar(0.905) More Information | Similar Motifs Found
6	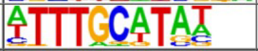	1e-19	-4.379e+01	5.05%	1.01%	51.5bp (61.6bp)	Oct2(POU,Homeobox)/Bcell-Oct2-ChIP-Seq(GSE21512)/Homer(0.955) More Information | Similar Motifs Found
7	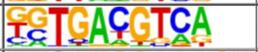	1e-18	-4.164e+01	14.43%	6.50%	55.2bp (62.6bp)	CREB1/MA0018.3/Jaspar(0.947) More Information | Similar Motifs Found
8	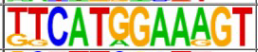	1e-15	-3.599e+01	1.11%	0.02%	38.1bp (42.7bp)	Bcl6/MA0463.1/Jaspar(0.730) More Information | Similar Motifs Found
9	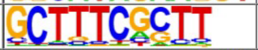	1e-14	-3.444e+01	4.34%	0.96%	47.1bp (72.3bp)	IRF2(IRF)/Erythroblas-IRF2-ChIP-Seq(GSE36985)/Homer(0.809) More Information | Similar Motifs Found
10	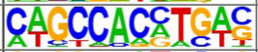	1e-14	-3.356e+01	6.05%	1.83%	56.7bp (58.0bp)	Creb3l2/MA0608.1/Jaspar(0.763) More Information | Similar Motifs Found
11	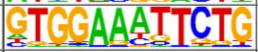	1e-13	-3.182e+01	3.73%	0.77%	49.8bp (61.3bp)	NFAT(RHD)/Jurkat-NFATC1-ChIP-Seq(Jolma_et_al.)/Homer(0.708) More Information | Similar Motifs Found
12	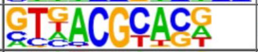	1e-13	-3.071e+01	4.84%	1.32%	51.1bp (66.0bp)	Atf1/MA0604.1/Jaspar(0.816) More Information | Similar Motifs Found
13	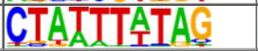	1e-13	-3.069e+01	3.33%	0.64%	55.6bp (63.5bp)	MEF2D/MA0773.1/Jaspar(0.953) More Information | Similar Motifs Found
14	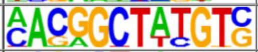	1e-12	-2.975e+01	0.61%	0.00%	66.6bp (41.6bp)	SOX10/MA0442.2/Jaspar(0.623) More Information | Similar Motifs Found
15	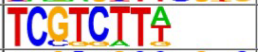	1e-12	-2.951e+01	8.07%	3.21%	53.2bp (65.9bp)	PB0108.1_Atf1_2/Jaspar(0.687) More Information | Similar Motifs Found

Rel 18h

Rank	Motif	P-value	log P-pvalue	% of Targets	% of Background	STD(Bg STD)	Best Match/Details
1	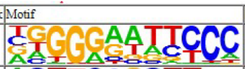	1e-276	-6.362e+02	19.92%	4.36%	49.9bp (62.8bp)	NFkB-p65(RHD)/GM12787-p65-ChIP-Seq(GSE19485)/Homer(0.917) More Information | Similar Motifs Found
2	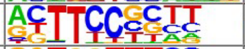	1e-254	-5.851e+02	31.04%	10.93%	53.4bp (62.7bp)	PB0058.1_Sfpi1_1/Jaspar(0.932) More Information | Similar Motifs Found
3	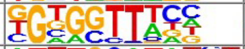	1e-115	-2.657e+02	20.79%	8.84%	54.3bp (63.4bp)	RUNX1(Runt)/Jurkat-RUNX1-ChIP-Seq(GSE29180)/Homer(0.844) More Information | Similar Motifs Found
4	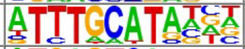	1e-62	-1.444e+02	4.75%	1.04%	51.6bp (59.6bp)	Oct11(POU,Homeobox)/NCIH1048-POU2F3-ChIP-seq(GSE115123)/Homer(0.979) More Information | Similar Motifs Found
5	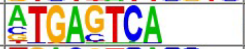	1e-52	-1.214e+02	14.04%	7.01%	55.4bp (62.8bp)	AP-1(bZIP)/ThioMac-PU.1-ChIP-Seq(GSE21512)/Homer(0.979) More Information | Similar Motifs Found
6	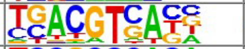	1e-50	-1.163e+02	13.96%	7.07%	55.2bp (62.3bp)	Atf1(bZIP)/K562-ATF1-ChIP-Seq(GSE31477)/Homer(0.913) More Information | Similar Motifs Found
7	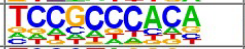	1e-40	-9.221e+01	24.26%	16.01%	55.7bp (63.2bp)	Egr2(Zf)/Thymocytes-Egr2-ChIP-Seq(GSE34254)/Homer(0.838) More Information | Similar Motifs Found
8	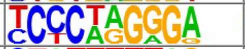	1e-38	-8.854e+01	6.12%	2.35%	52.6bp (58.2bp)	EBF(EBF)/proBcell-EBF-ChIP-Seq(GSE21978)/Homer(0.946) More Information | Similar Motifs Found
9	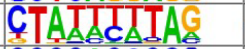	1e-30	-6.946e+01	4.21%	1.50%	52.1bp (63.2bp)	MEF2A/MA0052.3/Jaspar(0.915) More Information | Similar Motifs Found
10	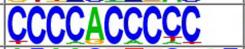	1e-27	-6.298e+01	12.77%	7.72%	53.6bp (65.5bp)	SP1/MA0079.3/Jaspar(0.882) More Information | Similar Motifs Found
11	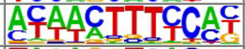	1e-25	-5.911e+01	0.36%	0.00%	54.9bp (32.1bp)	NFAT(RHD)/Jurkat-NFATC1-ChIP-Seq(Jolma_et_al.)/Homer(0.767) More Information | Similar Motifs Found
12	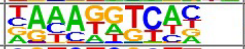	1e-25	-5.898e+01	9.31%	5.18%	55.1bp (64.5bp)	Nur77(NR)/K562-NR4A1-ChIP-Seq(GSE31363)/Homer(0.834) More Information | Similar Motifs Found
13	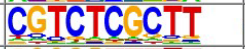	1e-24	-5.682e+01	10.05%	5.81%	57.4bp (65.4bp)	PAX5/MA0014.3/Jaspar(0.674) More Information | Similar Motifs Found
14	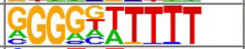	1e-20	-4.703e+01	3.40%	1.34%	52.5bp (72.2bp)	MF0003.1_REL_class/Jaspar(0.668) More Information | Similar Motifs Found
15	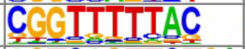	1e-20	-4.616e+01	3.27%	1.28%	58.0bp (67.4bp)	FOXI1/MA0042.2/Jaspar(0.704) More Information | Similar Motifs Found
16	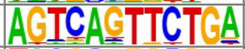	1e-17	-3.960e+01	0.56%	0.04%	62.0bp (49.7bp)	ZNF528(Zf)/HEK293-ZNF528.GFP-ChIP-Seq(GSE58341)/Homer(0.678) More Information | Similar Motifs Found
17	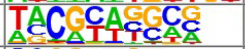	1e-14	-3.435e+01	1.83%	0.61%	56.2bp (58.5bp)	Gmeb1/MA0615.1/Jaspar(0.686) More Information | Similar Motifs Found
18	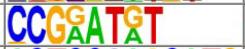	1e-12	-2.935e+01	1.75%	0.63%	50.2bp (60.1bp)	SPDEF/MA0686.1/Jaspar(0.880) More Information | Similar Motifs Found
19	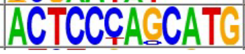	1e-12	-2.852e+01	0.25%	0.01%	54.7bp (71.9bp)	PB0206.1_Zic2_2/Jaspar(0.730) More Information | Similar Motifs Found

h

TFmotifView

http://bardet.u-strasbg.fr/tfmotifview/

RelA 1h

Motif ID	Number of target regions		Number of local control regions		Number of global control regions		Percent of regions with motifs			Enrichment over local controls		Enrichment over global controls	
	With motifs	Total	With motifs	Total	With motifs	Total	Target regions	Local controls	Global controls	Fold change	P-value	Fold change	P-value
ERG.MA0474.2	2170	3821	1388	3821	1333	3821	56.79%	36.33%	34.89%	1.5634	1.524e-72	1.6279	4.485e-83
REL.MA0101.1	2918	3821	2417	3821	2360	3821	76.37%	63.26%	61.76%	1.2073	3.918e-36	1.2364	8.004e-44
NFKB2.MA0778.1	421	3821	122	3821	122	3821	11.02%	3.19%	3.19%	3.4508	1.605e-42	3.4508	1.605e-42
ZBTB14.MA1650.1	1879	3821	1544	3821	1310	3821	49.18%	40.41%	34.28%	1.217	7.456e-15	1.4344	4.142e-40
GABPA.MA0062.3	2981	3821	2571	3821	2503	3821	78.02%	67.29%	65.51%	1.1595	3.425e-26	1.191	2.562e-34
AHR_ARNT.MA0006.1	2230	3821	1909	3821	1707	3821	58.36%	49.96%	44.67%	1.1682	9.839e-14	1.3064	2.654e-33
NRF1.MA0506.1	1011	3821	664	3821	621	3821	26.46%	17.38%	16.25%	1.5226	4.209e-22	1.628	5.769e-28
HES1.MA1099.2	1879	3821	1619	3821	1411	3821	49.18%	42.37%	36.93%	1.1606	1.348e-9	1.3317	1.654e-27
ZFP57.MA1583.1	1421	3821	1049	3821	983	3821	37.19%	27.45%	25.73%	1.3546	5.101e-20	1.4456	1.964e-27
HINFP.MA0131.2	1009	3821	650	3821	645	3821	26.41%	17.01%	16.88%	1.5523	1.089e-23	1.5643	2.348e-24

Rel 18h

Motif ID	Number of target regions		Number of local control regions		Number of global control regions		Percent of regions with motifs			Enrichment over local controls		Enrichment over global controls	
	With motifs	Total	With motifs	Total	With motifs	Total	Target regions	Local controls	Global controls	Fold change	P-value	Fold change	P-value
ERG.MA0474.2	1620	3940	962	3940	752	3940	41.12%	24.42%	19.09%	1.684	9.117e-57	2.1543	1.199e-102
REL.MA0101.1	2449	3940	1762	3940	1651	3940	62.16%	44.72%	41.90%	1.3899	1.04e-54	1.4833	5.269e-73
NFKB2.MA0778.1	356	3940	74	3940	67	3940	9.04%	1.88%	1.70%	4.8108	6.343e-48	5.3134	2.137e-51
GABPA.MA0062.3	2414	3940	1967	3940	1780	3940	61.27%	49.92%	45.18%	1.2272	2.173e-24	1.3562	7.866e-47
RELB.MA1117.1	2844	3940	2379	3940	2251	3940	72.18%	60.38%	57.13%	1.1955	8.037e-29	1.2634	8.878e-45
EGR1.MA0162.4	2511	3940	2272	3940	1969	3940	63.73%	57.66%	49.97%	1.1052	1.994e-8	1.2753	3.271e-35
ZBTB14.MA1650.1	1617	3940	1364	3940	1124	3940	41.04%	34.62%	28.53%	1.1855	2.367e-9	1.4386	1.016e-31
WT1.MA1627.1	2109	3940	1842	3940	1599	3940	53.53%	46.75%	40.58%	1.145	1.016e-9	1.3189	6.291e-31
AHR_ARNT.MA0006.1	1733	3940	1449	3940	1266	3940	43.98%	36.78%	32.13%	1.196	3.997e-11	1.3689	1.26e-27
KLF5.MA0599.1	2458	3940	2106	3940	1991	3940	62.39%	53.45%	50.53%	1.1671	5.501e-16	1.2346	1.471e-26

RelA 1h:
http://bardet.u-strasbg.fr/tfmotifview/?results=734fKxPAnCh8w1

Rel 18h:
http://bardet.u-strasbg.fr/tfmotifview/?results=H1bHoIdGkHrKPy

i

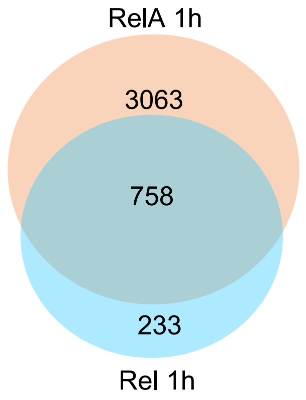

j

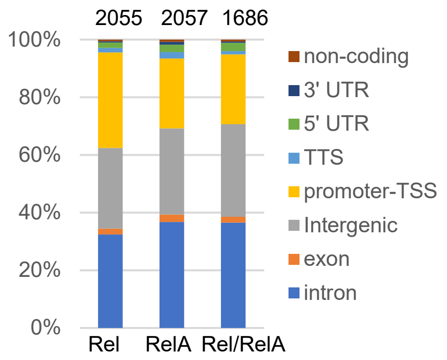

Figure S1

k

Rel-specific (2055)

Rank	Motif	P-value	log P-pvalue	% of Targets	% of Background	STD(Bg STD)	Best Match/Details
1	ACTTCCGCTT	1e-118	-2.736e+02	29.02%	10.45%	55.1bp (62.9bp)	PB0058.1_Sfpi1_1/Jaspar(0.963) More Information | Similar Motifs Found
2	GGGAATTCCCCA	1e-87	-2.021e+02	12.56%	2.78%	49.1bp (62.5bp)	NFkB-p65(RHD)/GM12787-p65-ChIP-Seq(GSE19485)/Homer(0.960) More Information | Similar Motifs Found
3	AACGGAAACT	1e-44	-1.036e+02	30.57%	17.72%	55.0bp (66.0bp)	IRF4(IRF)/GM12878-IRF4-ChIP-Seq(GSE32465)/Homer(0.901) More Information | Similar Motifs Found
4	ATTTGCATAATA	1e-33	-7.813e+01	4.48%	0.90%	51.0bp (60.7bp)	Oct11(POU,Homeobox)/NCIH1048-POU2F3-ChIP-seq(GSE115123)/Homer(0.961) More Information | Similar Motifs Found
5	TCCGCCCACA	1e-31	-7.341e+01	40.94%	28.70%	54.0bp (63.2bp)	Sp5(Zf)/mES-Sp5.Flag-ChIP-Seq(GSE72989)/Homer(0.836) More Information | Similar Motifs Found
6	ATGAGTCA	1e-26	-6.153e+01	12.90%	6.33%	54.5bp (61.5bp)	Fra1(bZIP)/BT549-Fra1-ChIP-Seq(GSE46166)/Homer(0.935) More Information | Similar Motifs Found
7	GTCACGTG	1e-22	-5.222e+01	9.01%	4.03%	52.8bp (65.9bp)	Arntl/MA0603.1/Jaspar(0.973) More Information | Similar Motifs Found
8	TAAAGGTCAC	1e-20	-4.665e+01	4.92%	1.66%	55.9bp (63.0bp)	NR4A1/MA1112.1/Jaspar(0.856) More Information | Similar Motifs Found
9	TGTGGTTTCGCA	1e-20	-4.618e+01	2.43%	0.46%	54.3bp (58.9bp)	RUNX1(Runt)/Jurkat-RUNX1-ChIP-Seq(GSE29180)/Homer(0.876) More Information | Similar Motifs Found
10	GGGCGTGG	1e-18	-4.213e+01	27.75%	19.64%	52.6bp (65.9bp)	SP3/MA0746.1/Jaspar(0.929) More Information | Similar Motifs Found
11	TTGCTGCACTAA	1e-15	-3.554e+01	0.39%	0.00%	54.7bp (0.0bp)	Zfp57(Zf)/H1-ZFP57.HA-ChIP-Seq(GSE115387)/Homer(0.647) More Information | Similar Motifs Found
12	TCACTTCT	1e-15	-3.541e+01	6.04%	2.69%	59.0bp (64.0bp)	PU.1-IRF(ETS:IRF)/Bcell-PU.1-ChIP-Seq(GSE21512)/Homer(0.816) More Information | Similar Motifs Found
13	GCGCATGCGC	1e-12	-2.933e+01	5.06%	2.28%	56.6bp (60.9bp)	NRF(NRF)/Promoter/Homer(0.972) More Information | Similar Motifs Found

RelA-specific (2057)

Rank	Motif	P-value	log P-pvalue	% of Targets	% of Background	STD(Bg STD)	Best Match/Details
1	GAAAGAGGAAGT	1e-58	-1.341e+02	14.39%	4.97%	52.9bp (59.9bp)	PB0058.1_Sfpi1_1/Jaspar(0.952) More Information | Similar Motifs Found
2	GGGATTTCCC	1e-35	-8.181e+01	24.65%	14.18%	54.2bp (62.7bp)	NFkB-p65(RHD)/GM12787-p65-ChIP-Seq(GSE19485)/Homer(0.924) More Information | Similar Motifs Found
3	AAAGCGGA	1e-22	-5.276e+01	39.67%	29.37%	56.6bp (64.9bp)	TFCP2/MA0145.3/Jaspar(0.747) More Information | Similar Motifs Found
4	TTATGCAAAT	1e-21	-5.018e+01	2.82%	0.57%	56.9bp (58.2bp)	Oct4(POU,Homeobox)/mES-Oct4-ChIP-Seq(GSE11431)/Homer(0.967) More Information | Similar Motifs Found
5	CACGTGAC	1e-19	-4.415e+01	13.22%	7.44%	54.0bp (62.4bp)	Arntl/MA0603.1/Jaspar(0.966) More Information | Similar Motifs Found
6	TCCCTGGGGAGT	1e-17	-4.070e+01	5.54%	2.20%	52.5bp (57.8bp)	EBF(EBF)/proBcell-EBF-ChIP-Seq(GSE21978)/Homer(0.982) More Information | Similar Motifs Found
7	ATGAGTCA	1e-15	-3.598e+01	6.81%	3.19%	55.5bp (60.0bp)	AP-1(bZIP)/ThioMac-PU.1-ChIP-Seq(GSE21512)/Homer(0.978) More Information | Similar Motifs Found
8	AAGCGAAA	1e-15	-3.534e+01	8.85%	4.66%	55.2bp (68.8bp)	IRF8(IRF)/BMDM-IRF8-ChIP-Seq(GSE77884)/Homer(0.865) More Information | Similar Motifs Found
9	TGCAACAGGGGG	1e-13	-3.097e+01	0.44%	0.01%	45.4bp (23.9bp)	SCRT2/MA0744.1/Jaspar(0.786) More Information | Similar Motifs Found
10	ATACCGCC	1e-13	-3.028e+01	14.29%	9.22%	57.3bp (61.9bp)	E2F3(E2F)/MEF-E2F3-ChIP-Seq(GSE71376)/Homer(0.658) More Information | Similar Motifs Found
11	TTTGGGGAAC	1e-12	-2.976e+01	2.77%	0.87%	56.7bp (60.7bp)	PB0162.1_Sfpi1_2/Jaspar(0.702) More Information | Similar Motifs Found
12	GCCACCATTTTG	1e-12	-2.954e+01	1.51%	0.28%	49.8bp (61.5bp)	YY1/MA0095.2/Jaspar(0.876) More Information | Similar Motifs Found
13	GTTTCTGATCTG	1e-12	-2.865e+01	1.17%	0.17%	43.8bp (63.8bp)	PB0033.1_Irf3_1/Jaspar(0.724) More Information | Similar Motifs Found
14	GGCAGCTTGCTA	1e-12	-2.848e+01	3.40%	1.26%	51.8bp (57.9bp)	ASCL1/MA1100.1/Jaspar(0.651) More Information | Similar Motifs Found

Rel/RelA shared (1686)

Rank	Motif	P-value	log P-pvalue	% of Targets	% of Background	STD(Bg STD)	Best Match/Details
1	GGGGAATTCC	1e-134	-3.099e+02	25.76%	6.55%	53.0bp (61.8bp)	NFkB-p65(RHD)/GM12787-p65-ChIP-Seq(GSE19485)/Homer(0.946) More Information | Similar Motifs Found
2	CACTTCCTCTTT	1e-108	-2.509e+02	29.55%	10.04%	52.1bp (61.4bp)	PB0058.1_Sfpi1_1/Jaspar(0.934) More Information | Similar Motifs Found
3	GAAGTGAAACTA	1e-64	-1.495e+02	8.55%	1.38%	54.1bp (69.4bp)	PU.1:IRF8(ETS:IRF)/pDC-Irf8-ChIP-Seq(GSE66899)/Homer(0.939) More Information | Similar Motifs Found
4	GAAAGCAC	1e-47	-1.096e+02	19.29%	8.07%	53.2bp (64.3bp)	IRF4(IRF)/GM12878-IRF4-ChIP-Seq(GSE32465)/Homer(0.798) More Information | Similar Motifs Found
5	AATTTGCATATG	1e-24	-5.552e+01	2.14%	0.20%	53.8bp (55.6bp)	Oct2(POU,Homeobox)/Bcell-Oct2-ChIP-Seq(GSE21512)/Homer(0.945) More Information | Similar Motifs Found
6	GTCCCTAGGG	1e-23	-5.450e+01	11.93%	5.48%	54.3bp (59.7bp)	EBF2(EBF)/BrownAdipose-EBF2-ChIP-Seq(GSE97114)/Homer(0.919) More Information | Similar Motifs Found
7	ATGACTCATT	1e-22	-5.152e+01	7.12%	2.53%	57.6bp (60.3bp)	JunB(bZIP)/DendriticCells-Junb-ChIP-Seq(GSE36099)/Homer(0.972) More Information | Similar Motifs Found
8	TAACCACAGG	1e-19	-4.575e+01	6.05%	2.09%	53.9bp (60.1bp)	RUNX(Runt)/HPC7-Runx1-ChIP-Seq(GSE22178)/Homer(0.939) More Information | Similar Motifs Found
9	CACGTGAC	1e-19	-4.514e+01	36.68%	26.46%	55.5bp (61.7bp)	USF1/MA0093.2/Jaspar(0.855) More Information | Similar Motifs Found
10	CTATATTTAG	1e-18	-4.161e+01	5.52%	1.92%	56.1bp (60.7bp)	MEF2A/MA0052.3/Jaspar(0.835) More Information | Similar Motifs Found
11	CACGCCCTGT	1e-12	-2.888e+01	1.84%	0.35%	46.8bp (64.1bp)	Klf12/MA0742.1/Jaspar(0.742) More Information | Similar Motifs Found

l

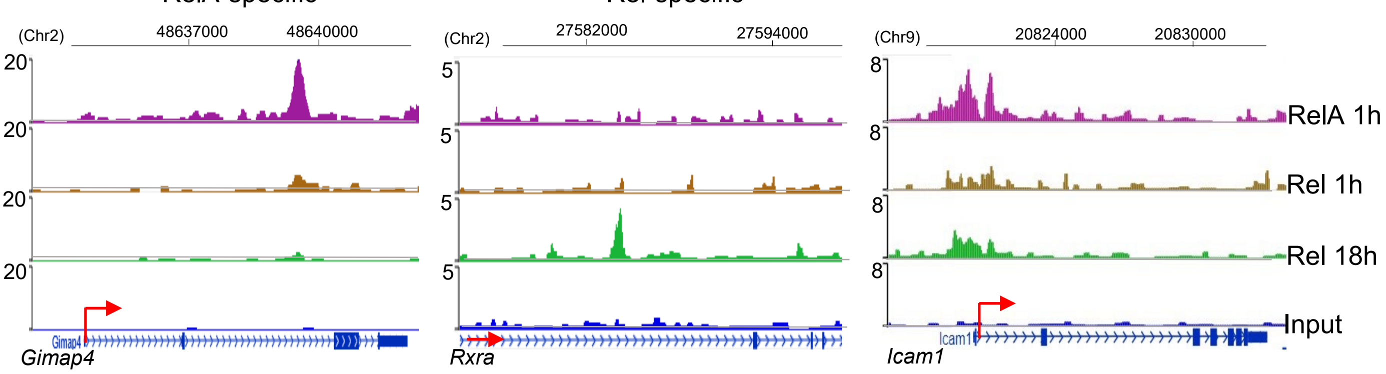

I continued..

Figure S1

RelA specific

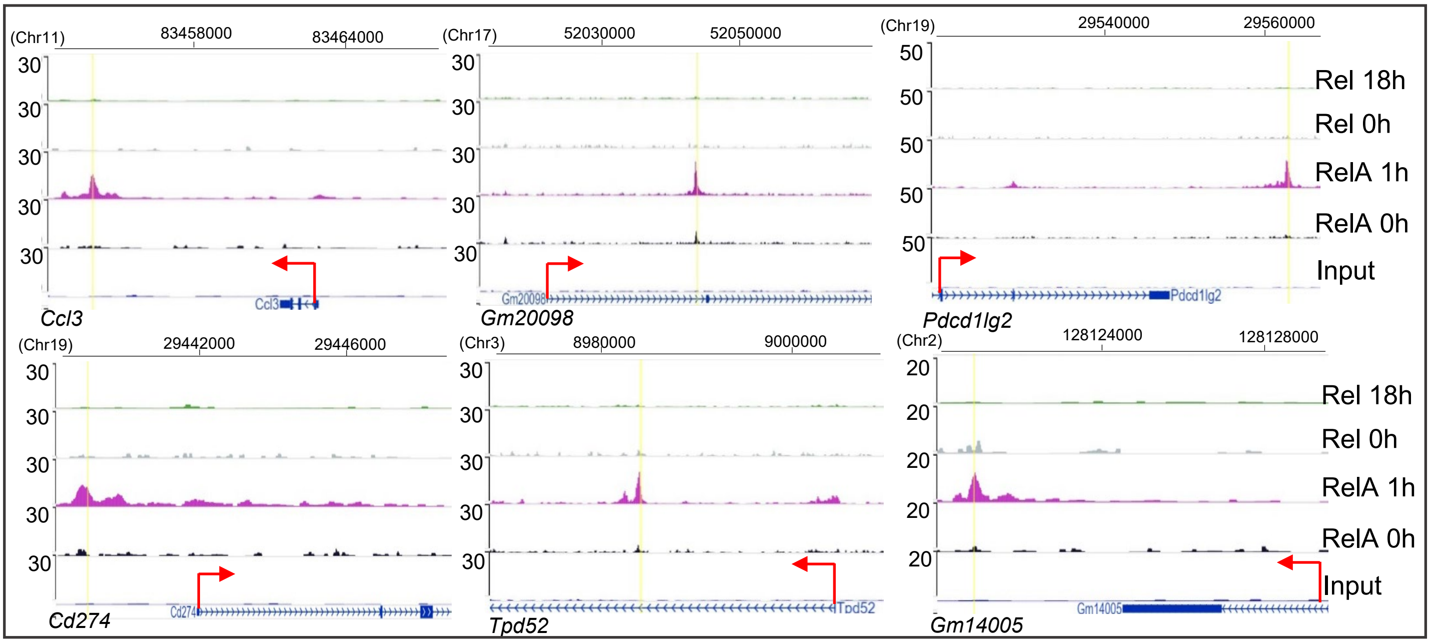

Rel specific

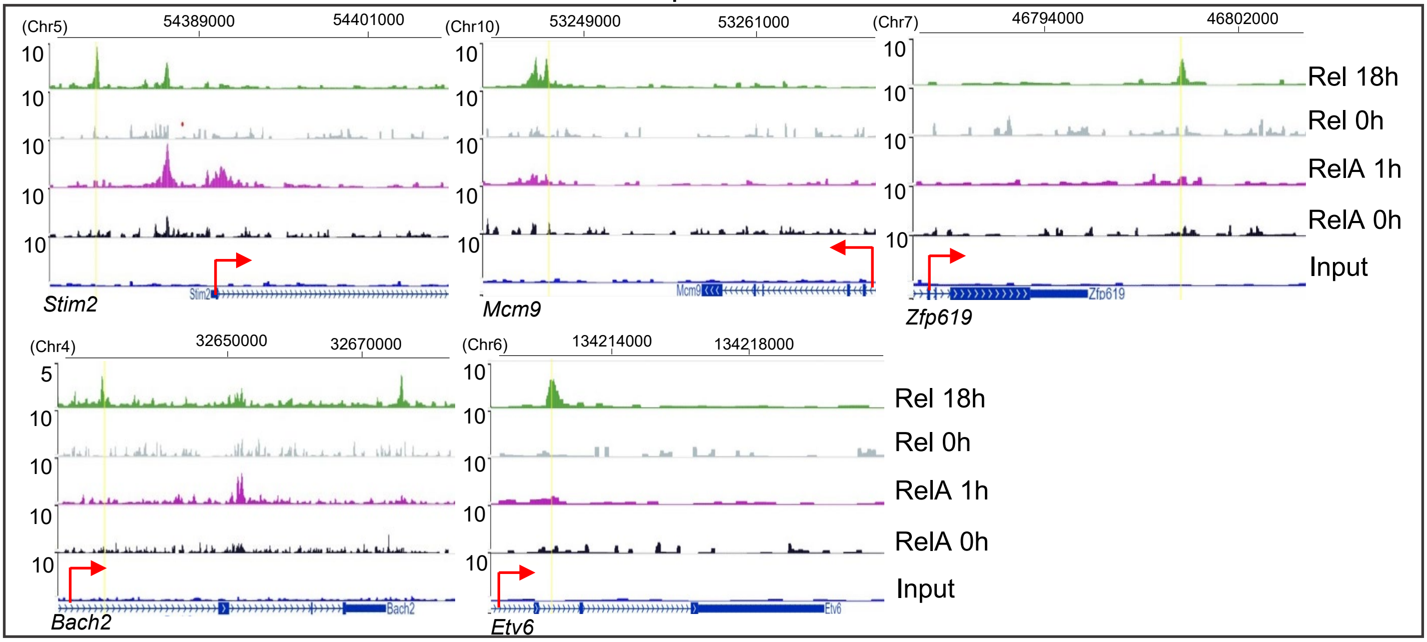

RelA/Rel shared

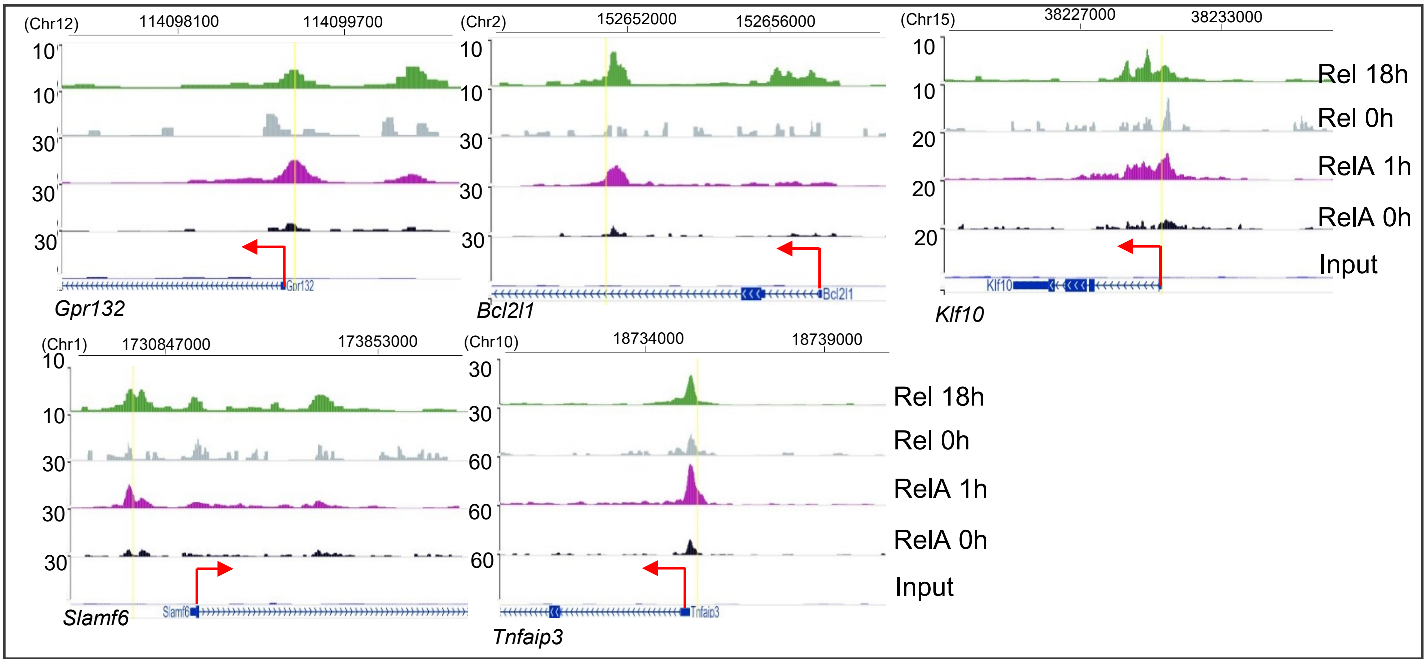

Figure S1: RelA and Rel ChIP-Seq in BCR activated spleen B cells

a, b RelA nuclear translocation after activation. Splenic B cells were activated with anti-IgM Fab`2 (10µg/ml) or PMA (50nM) + ionomycin (0.5µg/ml) for 1h and stained for RelA (RelA-APC) and nucleus (DAPI). RelA nuclear translocation was measured using Amnis Imagestream Mk II and quantified using IDEAS software **a,** Representative images of Imagestream at indicated times and activation regimes. Similarity index scores (yellow fonts) overlap between DAPI and RelA-APC channels **b,** Histogram representing RelA nuclear translocation (similarity index) of >6000 cells at (top) and bar graph representing mean of RelA translocation from 3 independent experiments (bottom). Error bars represent SEM. Translocation gate in the histogram indicates cells with nuclear RelA. **c,** Specificity check of anti-Rel and anti-RelA antibodies. RelA or Rel recruitment to promoter regions of target genes, WT and *Rel* KO splenic B cells activated as indicated. Fold change was calculated by comparison with an equal amount of input DNA (200pg) and a non-NF-κB binding genomic region. Error bar represents the SEM. *t*- test significance ** = $p \leq 0.005$. **d,** Bowtie analysis summary table of each replicate RelA and Rel ChIP-Seq library **e,** Scatterplots, created with deepTools bamCorrelate, depicting Pearson correlation between biological replicate RelA and Rel ChIP-Seq libraries **f, g** Complete list of significant transcription factor binding motifs (default statistical setting provided by HOMER) to enriched within RelA peaks **(f)**, Rel peaks **(g)** at different time points identified using HOMER (*de novo*). **h,** Transcription factors (TF) motifs under RelA 1h and Rel 18h peaks were identified by TFmotifView. The top 10 enriched over global control TF binding motifs are shown (statistical test provided according to TFmotifView). The entire TF binding motif analysis' can be found using the links provided. **i,** Venn diagram depicting the overlap between RelA and Rel binding sites genome-wide after 1h BCR crosslinking. Peaks were chosen with a threshold of 10-fold (RelA) and 5- fold (Rel) change compared to input. **j,** Genomic annotation of unique and shared Rel- and RelA-specific binding region (Figure 1b) using HOMER. **k,** List of significant transcription factor binding motifs (default statistical setting from HOMER) enriched within RelA-specific, Rel-specific and RelA/Rel shared peaks identified using HOMER (*de novo).* **l,** Representative genome browser tracks (mm9) of RelA-specific, Rel-specific and Rel/RelA shared binding of RelA at indicated times compared to input. The y axis represents normalized reads per 10 million.

a

Sample_name	Total reads	Overall alignment rate
H3K4me3 0h1	25211666	96.59%
H3K4me3 0h2	23659972	96.49%
H3K4me3 1h1	21764483	94.29%
H3K4me3 1h2	21925488	92.27%
H3K4me3 18h1	17167855	92.54%
H3K4me3 18h2	29715723	93.92%
H3K27ac 0h1	37095861	97.65%
H3K27ac 0h2	36375511	97.50%
H3K4me1 0h1	36185201	96.83%
H3K4me1 0h2	43800808	97.00%
ATAC 0h1	88994694	89.65%
ATAC 0h2	154175378	90.88%
ATAC 1h1	177728362	91.82%
ATAC 1h2	93133477	87.99%
ATAC 4h1	161913062	91.28%
ATAC 4h2	138408887	90.84%
ATAC 18h1	179624575	77.58%
ATAC 18h2	158563820	72.92%

b

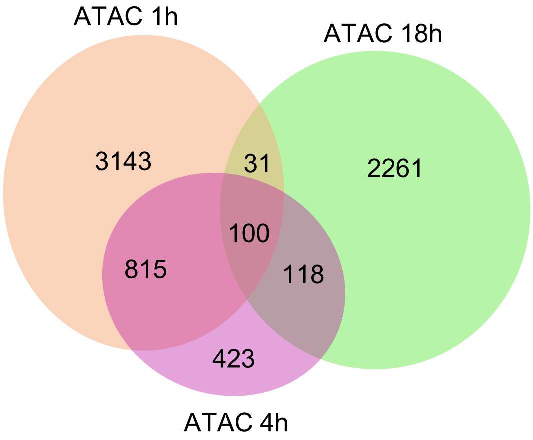

c

1h inducible ATAC peaks (*de novo*)

Consensus sequence	P-value	Motif Name
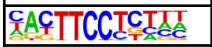	1e-190	Sfpi1
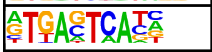	1e-167	Fra1(bZIP)
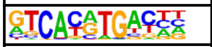	1e-103	TFE3(bHLH)
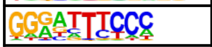	1e-102	NFkB-p65

18h inducible ATAC peaks (*de novo*)

Consensus sequence	P-value	Motif Name
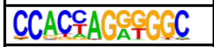	1e-83	BORIS(Zf)
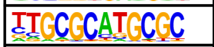	1e-25	NRF1(NRF)
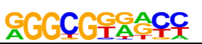	1e-23	Sp2(Zf)
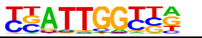	1e-23	POL004.1

d

1h unchanged ATAC peaks (*de novo*)

Consensus sequence	P-value	Motif Name
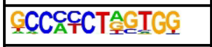	1e-960	BORIS(Zf)
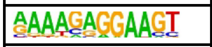	1e-360	SPIC
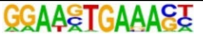	1e-178	PU.1:IRF8
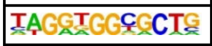	1e-77	CTCFL

18h unchanged ATAC peaks (*de novo*)

Consensus sequence	P-value	Motif Name
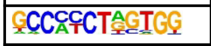	1e-491	BORIS(Zf)
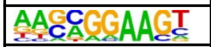	1e-117	Elf4(ETS)
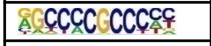	1e-104	Sp1(Zf)
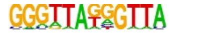	1e-77	ZNF652

Figure S2: Kinetics of chromatin accessibility and histone modifications in activated B cells.

a, Bowtie analysis. Table reporting Sample name, Total reads, and Overall alignment rate of each biological replicate ChIP-Seq and ATAC-Seq library. **b,** Venn diagram of the overlap of induced ATAC-seq peaks at 1h, 4h and 18h. Induced peaks were identified, using Diffbind settings of FDR$\leq$0.05 and fold change $\geq$1.5 compared with ATAC-seq at 0h. **c,** Motif analysis of sequences underlying induced ATAC-seq peaks compared to 0h was carried out using HOMER. The top 4 *de novo* motifs associated with differential ATAC-Seq peaks at 1h and 18h are shown. **d,** Motif analysis of sequences underlying ATAC-Seq peaks whose intensity did not change with BCR crosslinking was carried out using HOMER. The top 4 *de novo* motifs at 1h and 18h are shown.

a

Sample_name	Total reads	Overall alignment rate
RelA$^{fl/fl}$ 0h1	52709768	76.57%
RelA$^{fl/fl}$ 0h2	51660032	77.57%
RelA$^{fl/fl}$ 1h1	54038778	75.48%
RelA$^{fl/fl}$ 1h2	59370298	76.57%
RelA$^{fl/fl}$ 4h1	51755497	78.98%
RelA$^{fl/fl}$ 4h2	61225427	79.61%
RelA$^{fl/fl}$ 18h1	53191016	81.99%
RelA$^{fl/fl}$ 18h2	53202127	79.98%
RelA cKO 0h1	60172856	75.06%
RelA cKO 0h2	51524671	77.12%
RelA cKO 1h1	48708742	73.76%
RelA cKO 1h2	55075342	75.79%
RelA cKO 4h1	65529355	75.71%
RelA cKO 4h2	55624647	72.32%
RelA cKO 18h1	56854065	79.80%
RelA cKO 18h2	53563852	78.40%
WT 0h1	45489853	52.81%
WT 0h2	47990047	57.03%
WT 1h1	46439931	60.71%
WT 1h2	46858768	58.31%
WT 4h1	51951065	58.98%
WT 4h2	45914819	60.60%
WT 18h1	41878859	70.86%
WT 18h2	45061051	67.28%
BAY 0h1	45553160	51.94%
BAY 0h2	43840510	62.54%
BAY 1h1	42685079	62.50%
BAY 1h2	50860425	49.46%
BAY 4h1	45405779	65.48%
BAY 4h2	49110887	64.38%
BAY 18h1	49241869	56.47%
BAY 18h2	44424315	68.84%
Rel KO 0h1	54293019	48.50%
Rel KO 0h2	48109867	55.99%
Rel KO 1h1	51083828	45.51%
Rel KO 1h2	51274238	43.46%
Rel KO 4h1	50733414	56.40%
Rel KO 4h2	48787522	52.29%
Rel KO 18h1	43733327	59.29%
Rel KO 18h2	44460601	64.81%

b

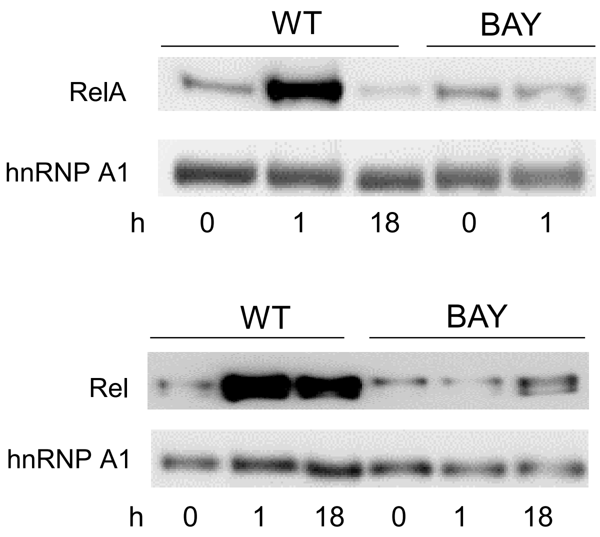

Figure S3

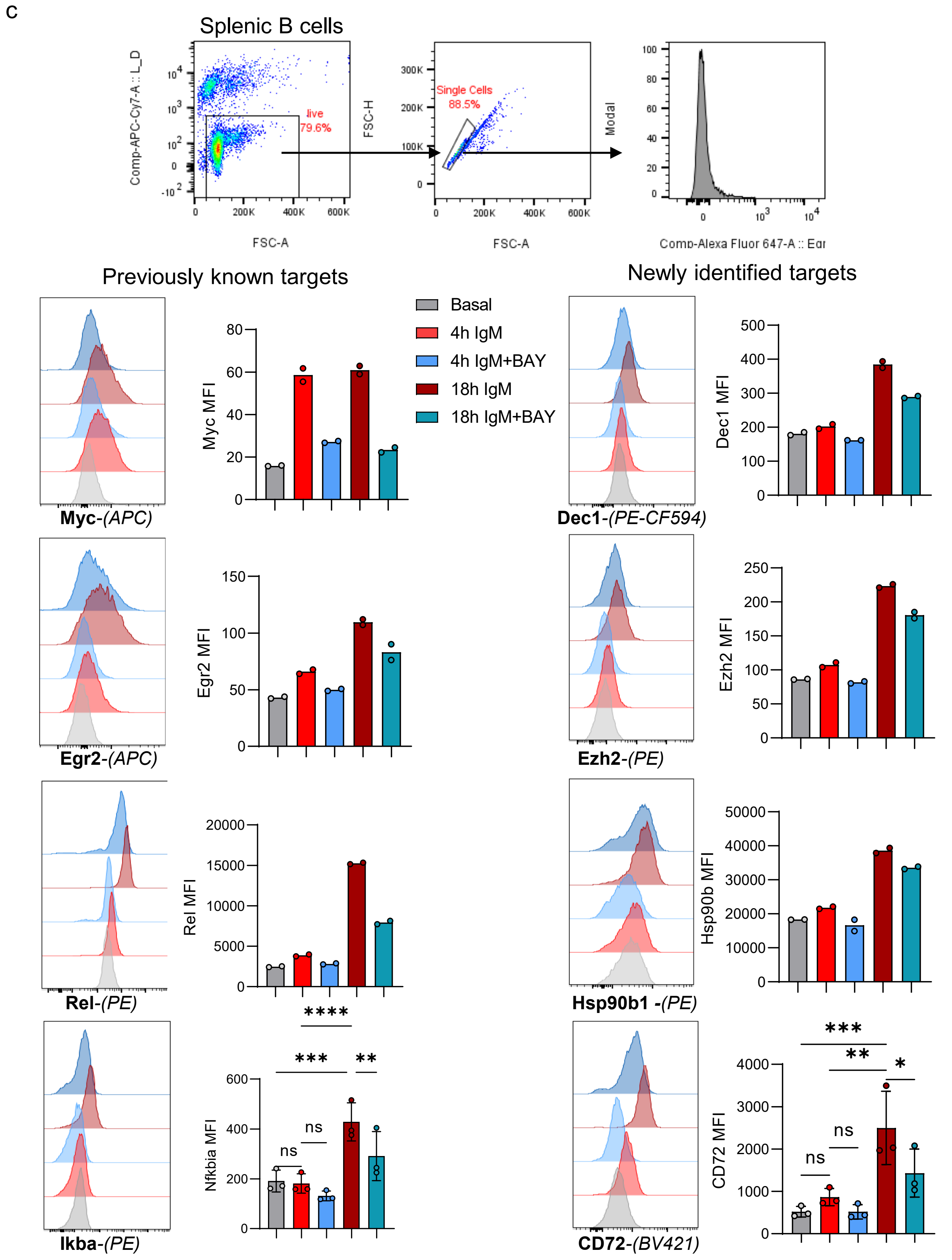
c
Splenic B cells
Comp-APC-Cy7-A :: L_D
live
79.6%
FSC-A
FSC-H
Single Cells
88.5%
FSC-A
Modal
Comp-Alexa Fluor 647-A :: Egr
Previously known targets
Newly identified targets
Basal
4h IgM
4h IgM+BAY
18h IgM
18h IgM+BAY
Myc MFI
Myc-(APC)
Egr2 MFI
Egr2-(APC)
Rel MFI
Rel-(PE)
Nfkbia MFI
Ikba-(PE)
Dec1 MFI
Dec1-(PE-CF594)
Ezh2 MFI
Ezh2-(PE)
Hsp90b MFI
Hsp90b1 -(PE)
CD72 MFI
CD72-(BV421)
****
***
**
*
ns

Figure S3

d

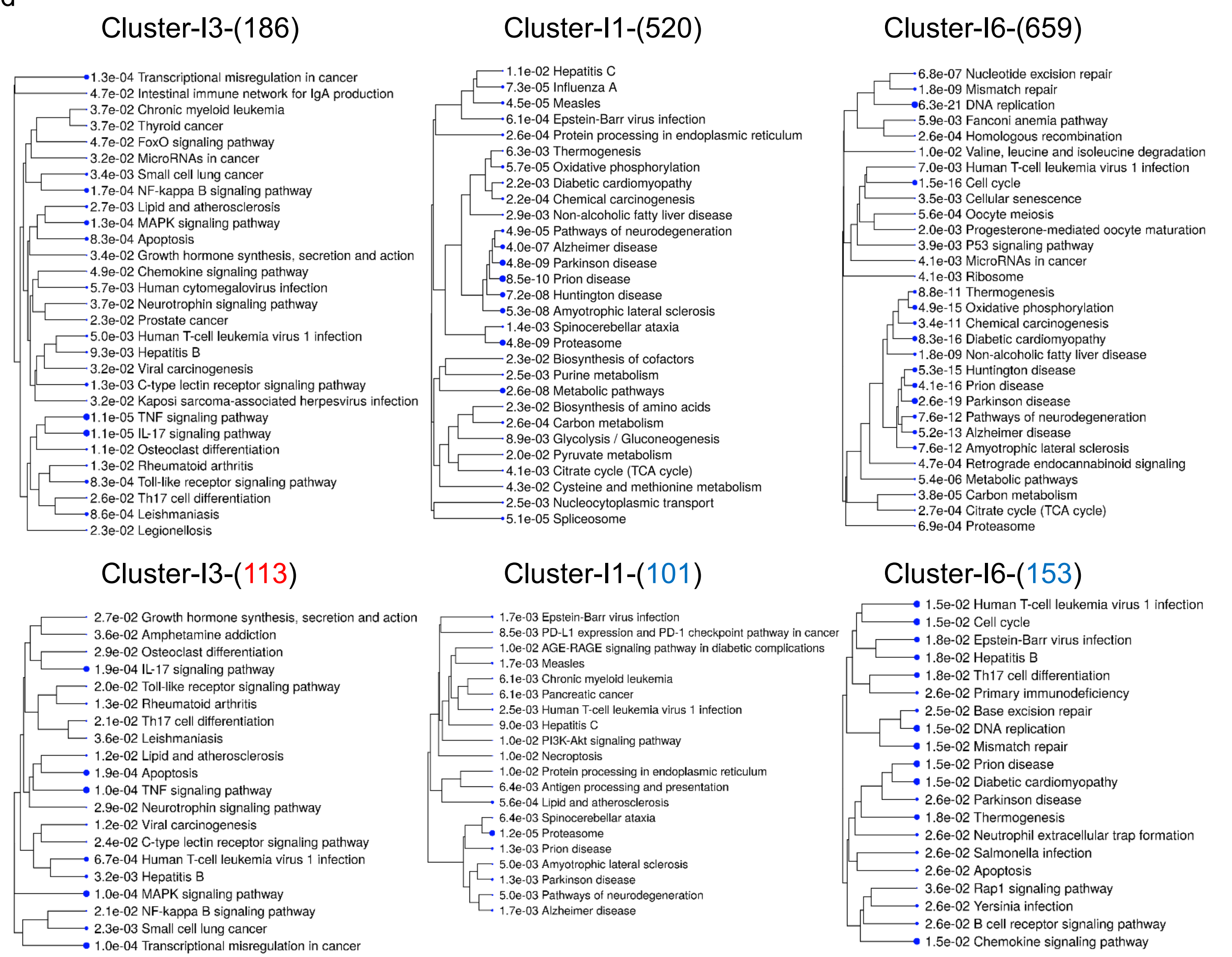

e

Upregulated 2-fold but not affected by inhibitor

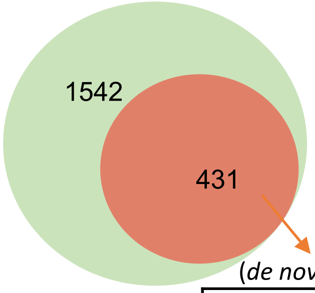

(*de novo*)

Consensus sequence	P-value	Motif Name
GGGAAATTCC	1e-21	NFkB-p65
CTCTCCAGTATG	1e-14	ZBTB26

Downregulated 2-fold but not affected by inhibitor

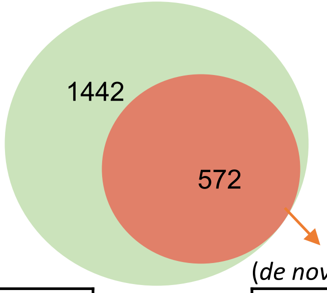

(*de novo*)

Consensus sequence	P-value	Motif Name
AGAGGAAGTG	1e-42	PU.1
GGGAAACCCCG	1e-37	NFkB-p65

f

Not changed in wild type

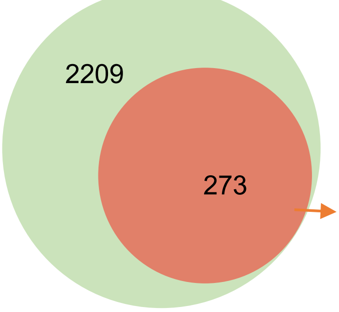

(*de novo*)

Consensus sequence	P-value	Motif Name
CGGAAGTG	1e-26	ETV6
GGAATTTCCC	1e-23	RELA
GGAAATCACCTT	1e-15	REL

Figure S3: Gene expression analyses in activated B cells.

a, Table of total reads and overall alignment rate, expressed as percentage of total, for 40 RNA-Seq samples including 20 biological replicates. RSEM was used to align samples to the mm9 reference genome. **b,** Kinetics of nuclear RelA and Rel induction in activated B cells in the presence or absence of the IKK2 inhibitor BAY11-7082. Nuclear extracts from splenic B cells treated with anti-IgM Fab`2, with or without inhibitor, for the indicated times (h) were probed with anti-RelA, anti-Rel or anti-hnRNP A1 (loading control) antibodies by western blot. Representative image of 3 experiments is shown. **c,** Gating strategy for identification of protein products of NF-κB target genes. Histograms and bar graphs representing mean of median fluorescence intensity (MFI) values are shown, left column known targets (Myc, Egr2, Rel: n=2, Ikba: n=3), right column newly identified NF-κB target genes (Dec1, Ezh2, Hsp90b1: n=2, CD72: n=3). For Ikba and CD72 error bar represent SEM and significance was measured using paired *t*-test, * ≤ 0.05, ** ≤ 0.01, *** ≤ 0.001 and **** ≤ 0.0001. **d,** KEGG pathways of gene clusters shown in Fig 3d. Top row – Hierarchically clustered pathways from all genes in cluster I3, I1 and I6. Bottom row - pathways from RelA- or Rel- binding genes of indicated clusters. Significant pathways were defined by an FDR of ≤ 0.05 - blues circle size in clusters indicates FDR values. **e,** Summary of NF-κB binding to genes whose inducible expression was unaffected by BAY11-7082. Top: 431 out of 1542 genes that were up-regulated ≥ 2x by anti-IgM Fab`2 treatment bound Rel or RelA. *De novo* motif analysis underlying these NF-κB peaks using HOMER is shown to the right of the Venn diagram. Bottom: 572 out of 1442 genes that were downregulated by anti-IgM Fab`2 treatment bound Rel or RelA; corresponding HOMER analysis is shown to the right, with default setting for p-values in HOMER. **f,** RNA-Seq data representing expression of 2209 genes that did not change with anti-IgM Fab`2 and inhibitor treatment. Of these, 273 showed inducible RelA or Rel binding (orange circle). HOMER analysis of sequences underlying these peaks is shown to the right.

Figure S4

a

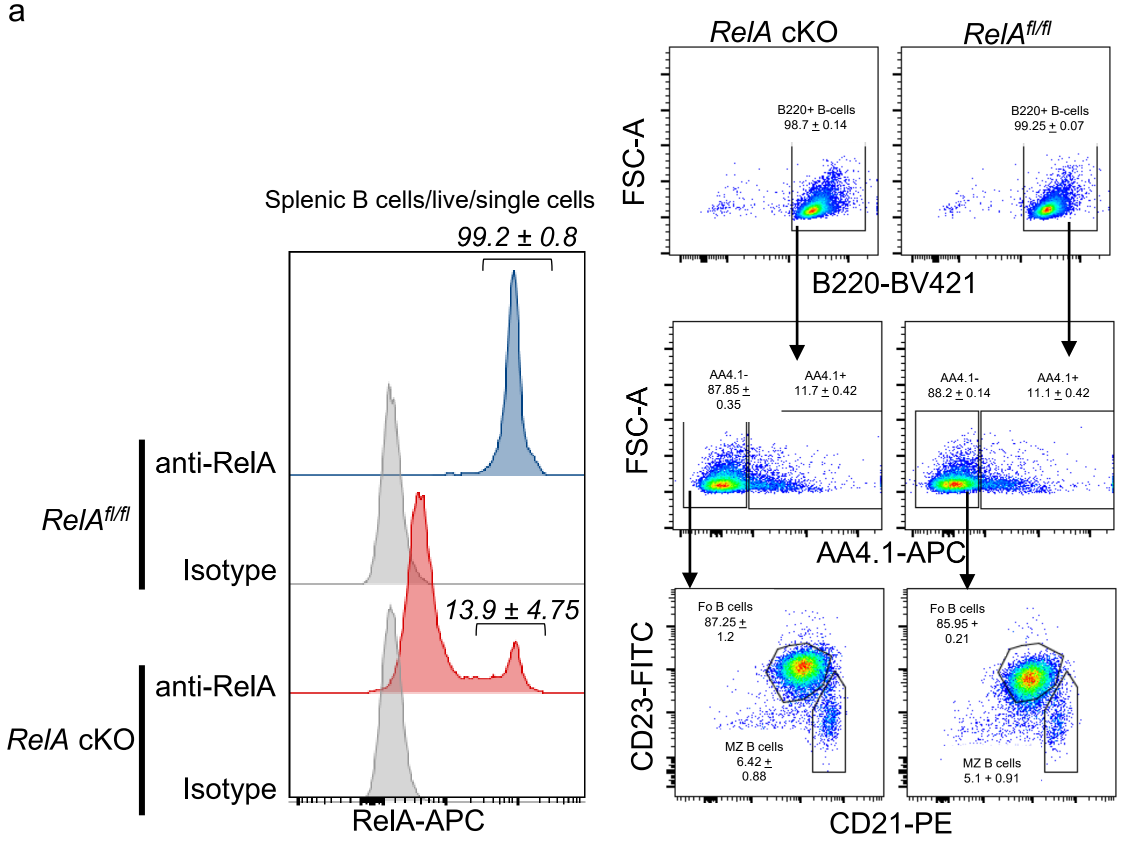

Splenic B cells/live/single cells
99.2 ± 0.8
13.9 ± 4.75
anti-RelA
Isotype
RelA^fl/fl
RelA cKO
RelA-APC
RelA cKO
RelA^fl/fl
FSC-A
B220+ B-cells
98.7 ± 0.14
B220+ B-cells
99.25 ± 0.07
B220-BV421
AA4.1-
87.85 ± 0.35
AA4.1+
11.7 ± 0.42
AA4.1-
88.2 ± 0.14
AA4.1+
11.1 ± 0.42
AA4.1-APC
CD23-FITC
Fo B cells
87.25 ± 1.2
MZ B cells
6.42 ± 0.88
Fo B cells
85.95 + 0.21
MZ B cells
5.1 + 0.91
CD21-PE

b

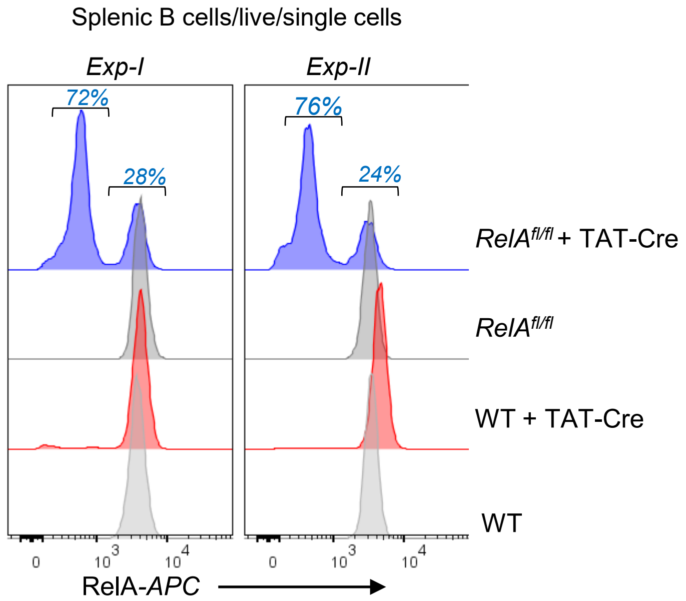

Splenic B cells/live/single cells
Exp-I
Exp-II
72%
28%
76%
24%
RelA^fl/fl + TAT-Cre
RelA^fl/fl
WT + TAT-Cre
WT
0
10^3
10^4
RelA-APC

c

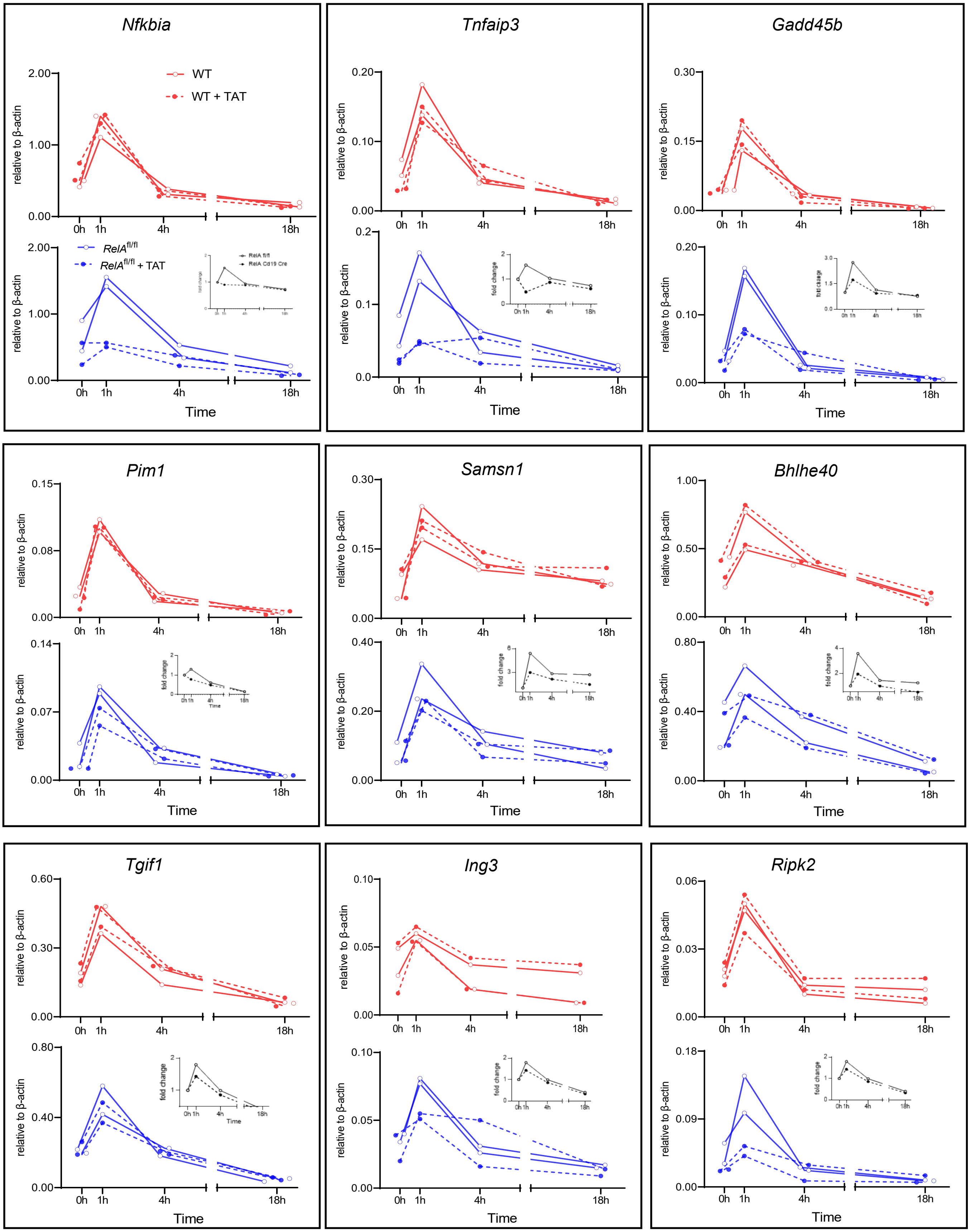
Nfkbia
Tnfaip3
Gadd45b
Pim1
Samsn1
Bhlhe40
Tgif1
Ing3
Ripk2
WT
WT + TAT
RelA fl/fl
RelA fl/fl + TAT
RelA fl/fl
RelA Cd19 Cre
relative to β-actin
fold change
0h
1h
4h
18h
Time

d

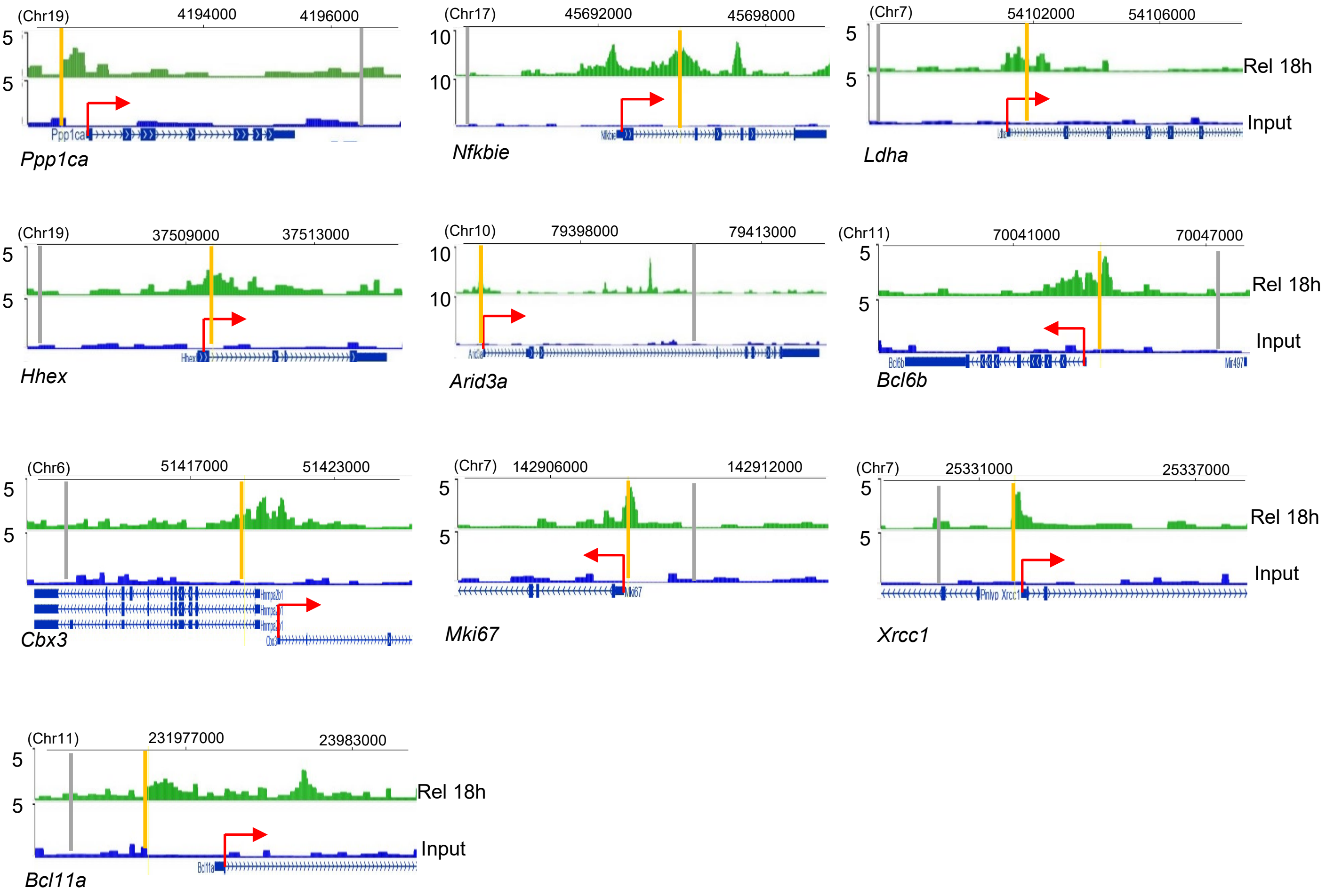
(Chr19) 4194000 4196000
5
5
Ppp1ca
Ppp1ca
(Chr17) 45692000 45698000
10
10
Nfkbie
(Chr7) 54102000 54106000
5
5
Rel 18h
Input
Ldha
(Chr19) 37509000 37513000
Hhex
Hhex
(Chr10) 79398000 79413000
Arid3a
(Chr11) 70041000 70047000
Rel 18h
Input
Bcl6b
Bcl6b
Mir497
(Chr6) 51417000 51423000
Cbx3
(Chr7) 142906000 142912000
Mki67
Mki67
(Chr7) 25331000 25337000
Rel 18h
Input
Xrcc1
(Chr11) 231977000 23983000
Rel 18h
Input
Bcl11a
Bcl11a

e

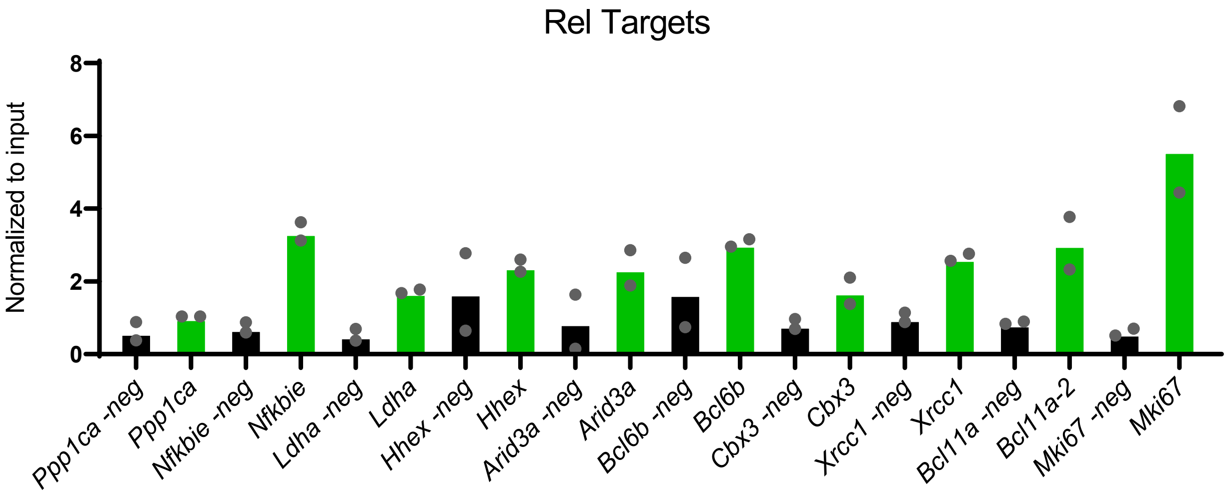
Rel Targets
Normalized to input
8
6
4
2
0
Ppp1ca -neg
Ppp1ca
Nfkbie -neg
Nfkbie
Ldha -neg
Ldha
Hhex -neg
Hhex
Arid3a -neg
Arid3a
Bcl6b -neg
Bcl6b
Cbx3 -neg
Cbx3
Xrcc1 -neg
Xrcc1
Bcl11a -neg
Bcl11a-2
Mki67 -neg
Mki67

Figure S4: Gene expression and ChIP analysis of RelA and Rel target genes.

a, Flow cytometry analysis of RelA expression in splenic B cells of *RelA cKO* and *RelA*$^{fl/fl}$ mice (left) and scatter plots depicting the distribution of typical splenic B cell subsets (Fo = Follicular B cells; MZ marginal zone B cells) identified by markers, B220, AA4.1, CD23 and CD21 (right) (n=2; number represent mean value ± STD). **b,** *Ex vivo* deletion of RelA in B cells using TAT-Cre. Histograms from 2 independent experiments representing RelA levels are shown. **c,** Gene expression of RelA selective genes in *ex vivo* RelA deleted B cells. TAT-Cre treated B cells of *RelA*$^{fl/fl}$ or C57BL/6 genotypes (Figure S4b) were stimulated with anti-IgM Fab`2 and qRT-PCR was done. Red graphs represent C57BL/6 (WT) and WT+TAT-Cre treated B cells; blue graphs represent *RelA*$^{fl/fl}$ and *RelA*$^{fl/fl}$ + TAT-Cre treated B cells. Black inset graphs represent the fold change values between *RelA*$^{fl/fl}$ and *RelA*$^{fl/fl}$ x *Cd19-cre* at 0, 1, 4 and 18h obtained from RNA-seq data shown in Figure 3e. Data for individual genes are represented as relative expression to β-actin (relative expression = $2^{(CT\ (\beta\text{-actin})-CT\ (\text{target gene}))}$). **d,** Representative browser tracks, based on mm9 annotation, of ChIP-Seq profiles of Rel target genes 18h after anti-IgM treatment. Yellow lines indicate the positive primer site, grey lines indicate negative control sites in each locus. The Y axis represents normalized reads per 10 million of aligned reads. **e,** Independent ChIP-PCR of Rel targets at 18h after anti-IgM treatment. Data shown are the average of 2 additional ChIP experiments, normalized to input value; grey bars represent negative control amplicons from Figure S4d.

Figure S5

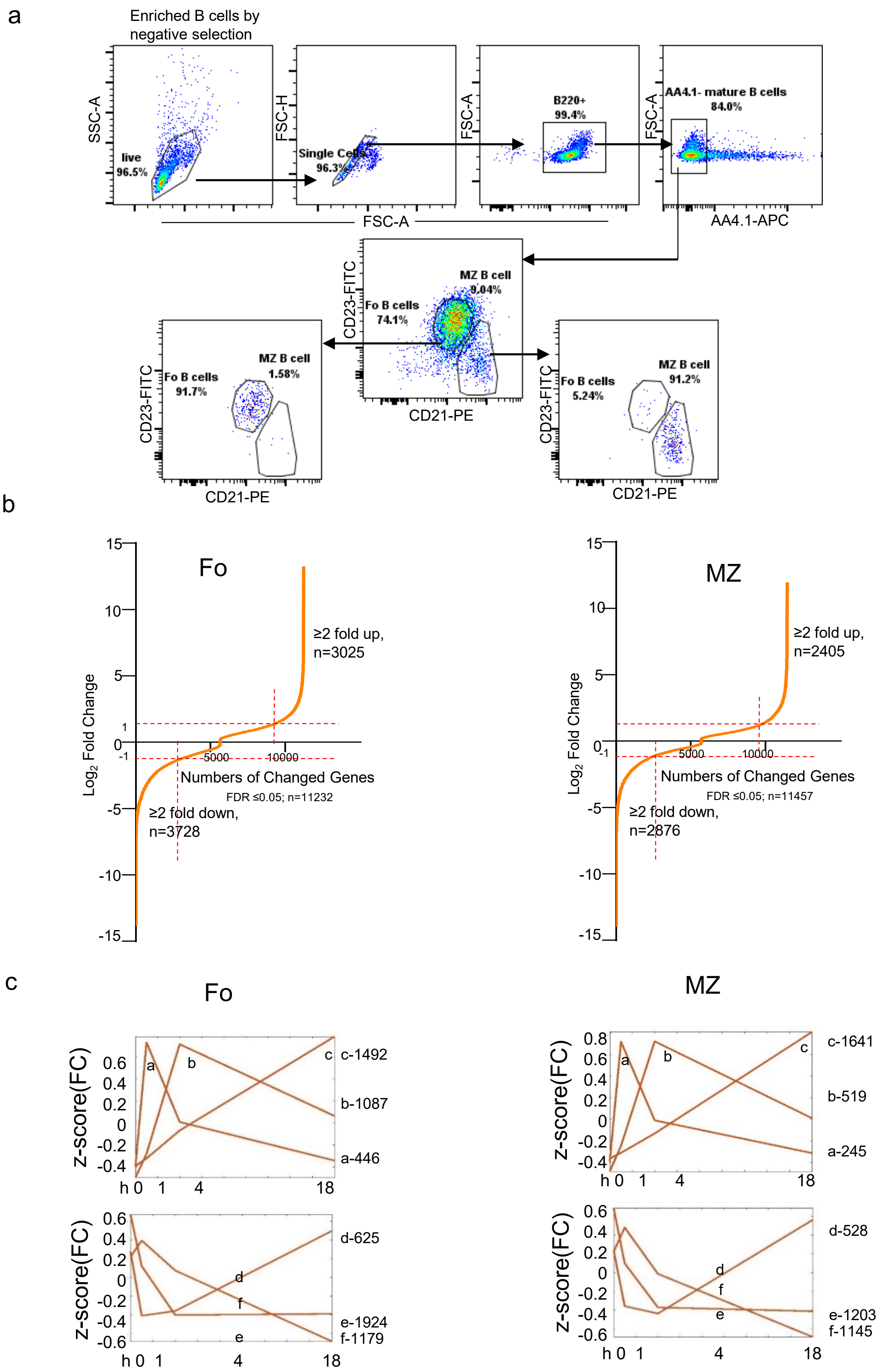
a
Enriched B cells by negative selection
SSC-A
live
96.5%
FSC-H
Single Cells
96.3%
FSC-A
B220+
99.4%
FSC-A
AA4.1- mature B cells
84.0%
FSC-A
AA4.1-APC
CD23-FITC
MZ B cell
9.04%
Fo B cells
74.1%
CD21-PE
CD23-FITC
Fo B cells
91.7%
MZ B cell
1.58%
CD21-PE
CD23-FITC
Fo B cells
5.24%
MZ B cell
91.2%
CD21-PE
b
Fo
Log2 Fold Change
≥2 fold up,
n=3025
≥2 fold down,
n=3728
Numbers of Changed Genes
FDR ≤0.05; n=11232
MZ
≥2 fold up,
n=2405
≥2 fold down,
n=2876
Numbers of Changed Genes
FDR ≤0.05; n=11457
c
Fo
z-score(FC)
c-1492
b-1087
a-446
d-625
e-1924
f-1179
MZ
c-1641
b-519
a-245
d-528
e-1203
f-1145
h 0 1 4 18

Figure S5

d

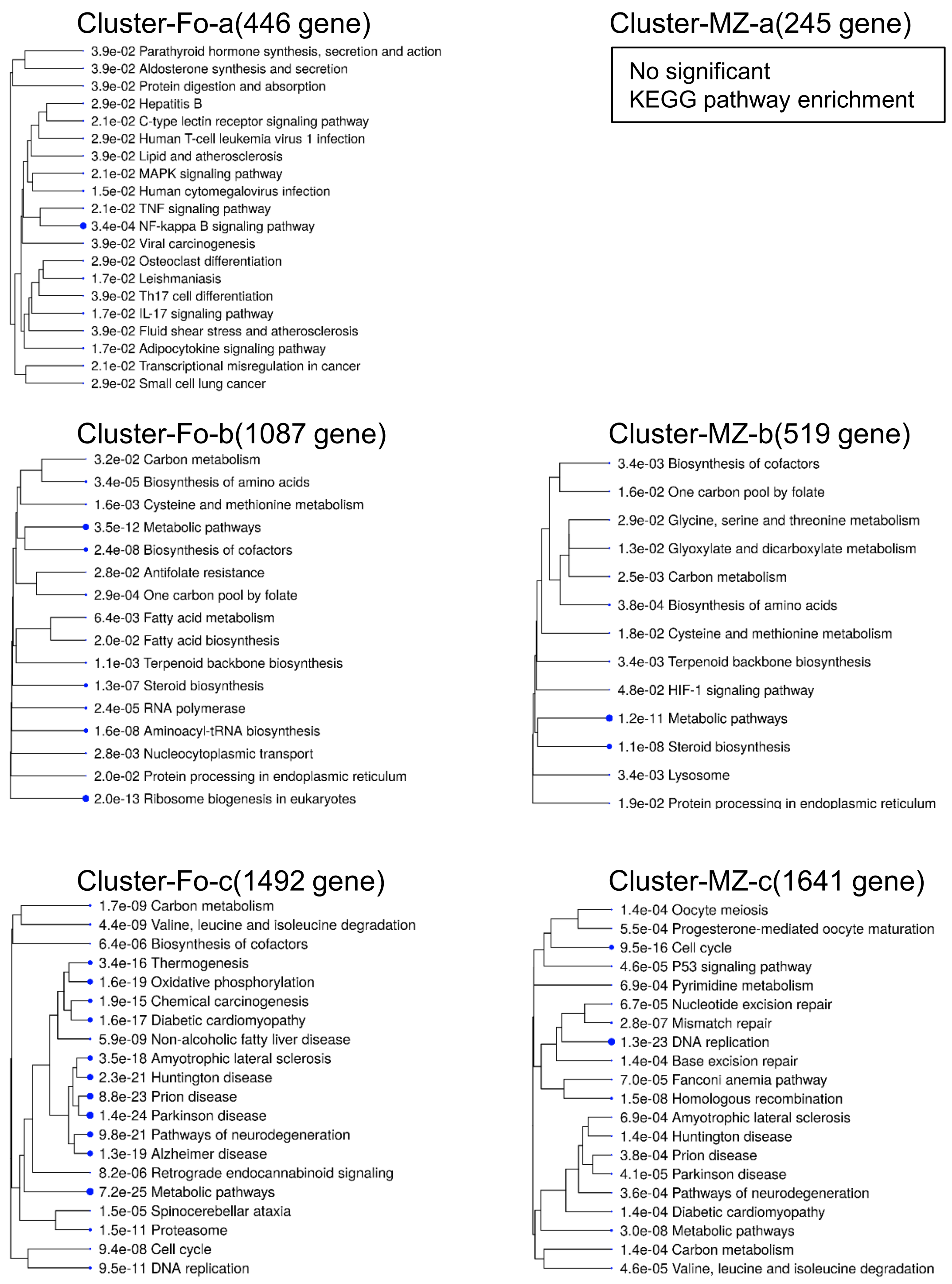
Cluster-Fo-a(446 gene)
3.9e-02 Parathyroid hormone synthesis, secretion and action
3.9e-02 Aldosterone synthesis and secretion
3.9e-02 Protein digestion and absorption
2.9e-02 Hepatitis B
2.1e-02 C-type lectin receptor signaling pathway
2.9e-02 Human T-cell leukemia virus 1 infection
3.9e-02 Lipid and atherosclerosis
2.1e-02 MAPK signaling pathway
1.5e-02 Human cytomegalovirus infection
2.1e-02 TNF signaling pathway
3.4e-04 NF-kappa B signaling pathway
3.9e-02 Viral carcinogenesis
2.9e-02 Osteoclast differentiation
1.7e-02 Leishmaniasis
3.9e-02 Th17 cell differentiation
1.7e-02 IL-17 signaling pathway
3.9e-02 Fluid shear stress and atherosclerosis
1.7e-02 Adipocytokine signaling pathway
2.1e-02 Transcriptional misregulation in cancer
2.9e-02 Small cell lung cancer
Cluster-MZ-a(245 gene)
No significant
KEGG pathway enrichment
Cluster-Fo-b(1087 gene)
3.2e-02 Carbon metabolism
3.4e-05 Biosynthesis of amino acids
1.6e-03 Cysteine and methionine metabolism
3.5e-12 Metabolic pathways
2.4e-08 Biosynthesis of cofactors
2.8e-02 Antifolate resistance
2.9e-04 One carbon pool by folate
6.4e-03 Fatty acid metabolism
2.0e-02 Fatty acid biosynthesis
1.1e-03 Terpenoid backbone biosynthesis
1.3e-07 Steroid biosynthesis
2.4e-05 RNA polymerase
1.6e-08 Aminoacyl-tRNA biosynthesis
2.8e-03 Nucleocytoplasmic transport
2.0e-02 Protein processing in endoplasmic reticulum
2.0e-13 Ribosome biogenesis in eukaryotes
Cluster-MZ-b(519 gene)
3.4e-03 Biosynthesis of cofactors
1.6e-02 One carbon pool by folate
2.9e-02 Glycine, serine and threonine metabolism
1.3e-02 Glyoxylate and dicarboxylate metabolism
2.5e-03 Carbon metabolism
3.8e-04 Biosynthesis of amino acids
1.8e-02 Cysteine and methionine metabolism
3.4e-03 Terpenoid backbone biosynthesis
4.8e-02 HIF-1 signaling pathway
1.2e-11 Metabolic pathways
1.1e-08 Steroid biosynthesis
3.4e-03 Lysosome
1.9e-02 Protein processing in endoplasmic reticulum
Cluster-Fo-c(1492 gene)
1.7e-09 Carbon metabolism
4.4e-09 Valine, leucine and isoleucine degradation
6.4e-06 Biosynthesis of cofactors
3.4e-16 Thermogenesis
1.6e-19 Oxidative phosphorylation
1.9e-15 Chemical carcinogenesis
1.6e-17 Diabetic cardiomyopathy
5.9e-09 Non-alcoholic fatty liver disease
3.5e-18 Amyotrophic lateral sclerosis
2.3e-21 Huntington disease
8.8e-23 Prion disease
1.4e-24 Parkinson disease
9.8e-21 Pathways of neurodegeneration
1.3e-19 Alzheimer disease
8.2e-06 Retrograde endocannabinoid signaling
7.2e-25 Metabolic pathways
1.5e-05 Spinocerebellar ataxia
1.5e-11 Proteasome
9.4e-08 Cell cycle
9.5e-11 DNA replication
Cluster-MZ-c(1641 gene)
1.4e-04 Oocyte meiosis
5.5e-04 Progesterone-mediated oocyte maturation
9.5e-16 Cell cycle
4.6e-05 P53 signaling pathway
6.9e-04 Pyrimidine metabolism
6.7e-05 Nucleotide excision repair
2.8e-07 Mismatch repair
1.3e-23 DNA replication
1.4e-04 Base excision repair
7.0e-05 Fanconi anemia pathway
1.5e-08 Homologous recombination
6.9e-04 Amyotrophic lateral sclerosis
1.4e-04 Huntington disease
3.8e-04 Prion disease
4.1e-05 Parkinson disease
3.6e-04 Pathways of neurodegeneration
1.4e-04 Diabetic cardiomyopathy
3.0e-08 Metabolic pathways
1.4e-04 Carbon metabolism
4.6e-05 Valine, leucine and isoleucine degradation

Figure S5

e

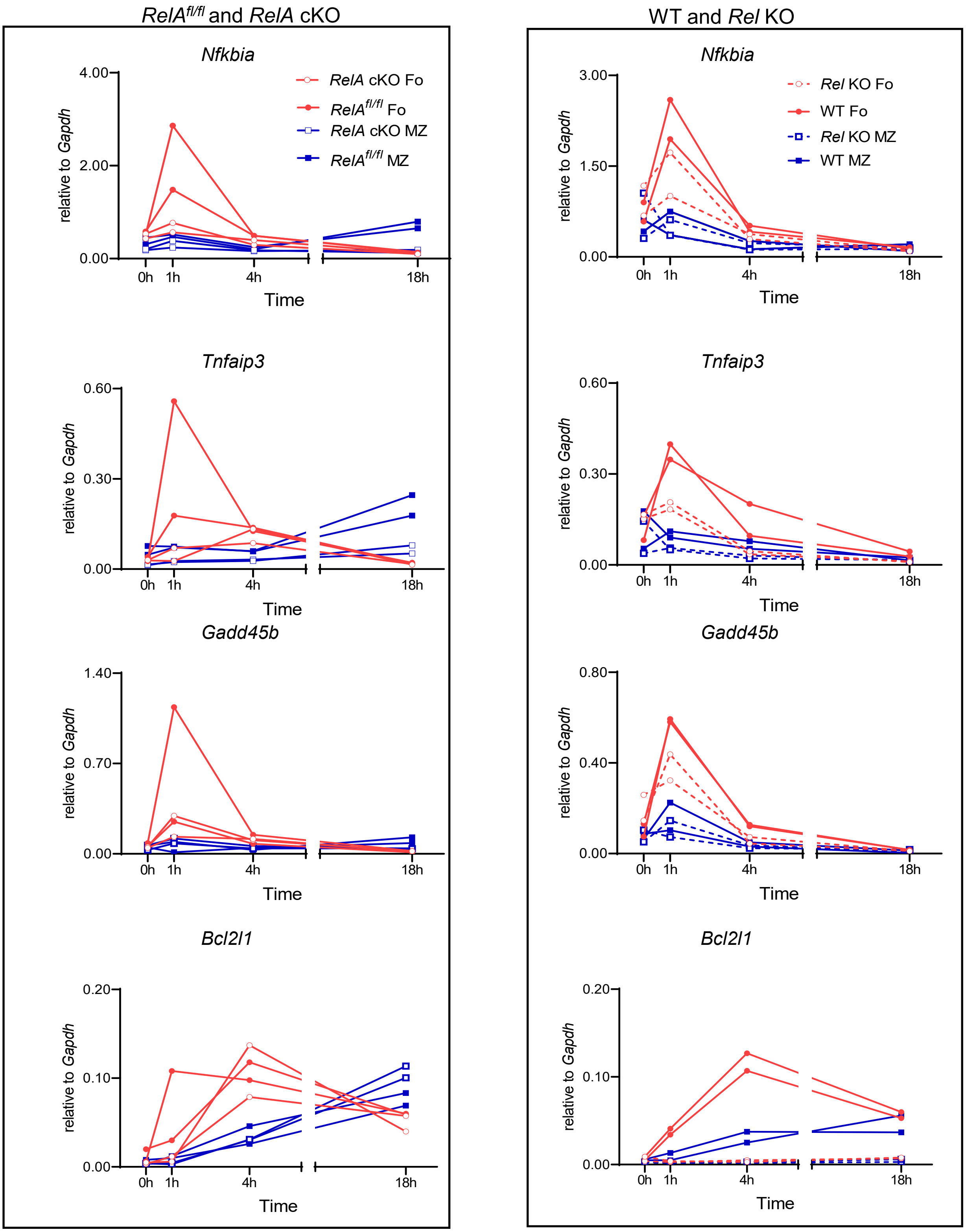

$RelA^{fl/fl}$ and $RelA$ cKO
WT and Rel KO
Nfkbia
Tnfaip3
Gadd45b
Bcl2l1
relative to *Gapdh*
Time
0h
1h
4h
18h
RelA cKO Fo
$RelA^{fl/fl}$ Fo
RelA cKO MZ
$RelA^{fl/fl}$ MZ
Rel KO Fo
WT Fo
Rel KO MZ
WT MZ
4.00
2.00
0.00
3.00
1.50
0.60
0.30
1.40
0.70
0.80
0.40
0.20
0.10

Figure S5: Gene expression in follicular (Fo) and marginal zone (MZ) B cells.

a, FACS sorting strategy for splenic Fo and MZ B cells isolation from negatively selected splenic B cells. Post sort purity is shown for Fo and Mz B cells (bottom panel). **b,** RNA-Seq was performed with RNA from Fo and MZ B cells activated with anti-IgM for 0, 1, 4 and 18h. Differential gene expression (using EBSeq - FDR$\leq$0.05) from two biological replicate experiments. Plot of log2 fold changes in gene expression at either 1, 4 or 18 h post activation compared to un-activated cells in Fo (left) and MZ (right) cells. Horizontal red dotted lines correspond to fold change thresholds of $\geq$ 2. Numbers of genes up- or down-regulated by $\geq$ 2x are indicated. **c,** In both Fo (left) and MZ (right) cells, inducible transcripts were partitioned into 3 up (a-c) and 3 down (d-f) regulated kinetic patterns by k-means clustering. Each graph represents the centroid profile of the average of z-score of fold change (FC) compared with untreated cells at 1, 4, and 18 h. Total numbers of genes in each pattern are indicated on the right of each panel. **d,** Enriched KEGG pathways from Fo and MZ B cell genes from indicated k-means clusters in Fig S5c. Similar pathways are represented by hierarchical clustering. FDR ≤ 0.05 qualifies for significant pathways and blues circle size in clusters indicates FDR values. **e,** Gene expression analysis by qPCR in Fo and MZ B cells of *RelA* cKO and *Rel* KO mice after anti-IgM stimulation. Relative to *Gapdh* expression levels of indicated genes are shown. Left panel represents *RelA* cKO compared with $RelA^{fl/fl}$ and right panel are *Rel* KO compared with WT, (n=2).

Figure S6

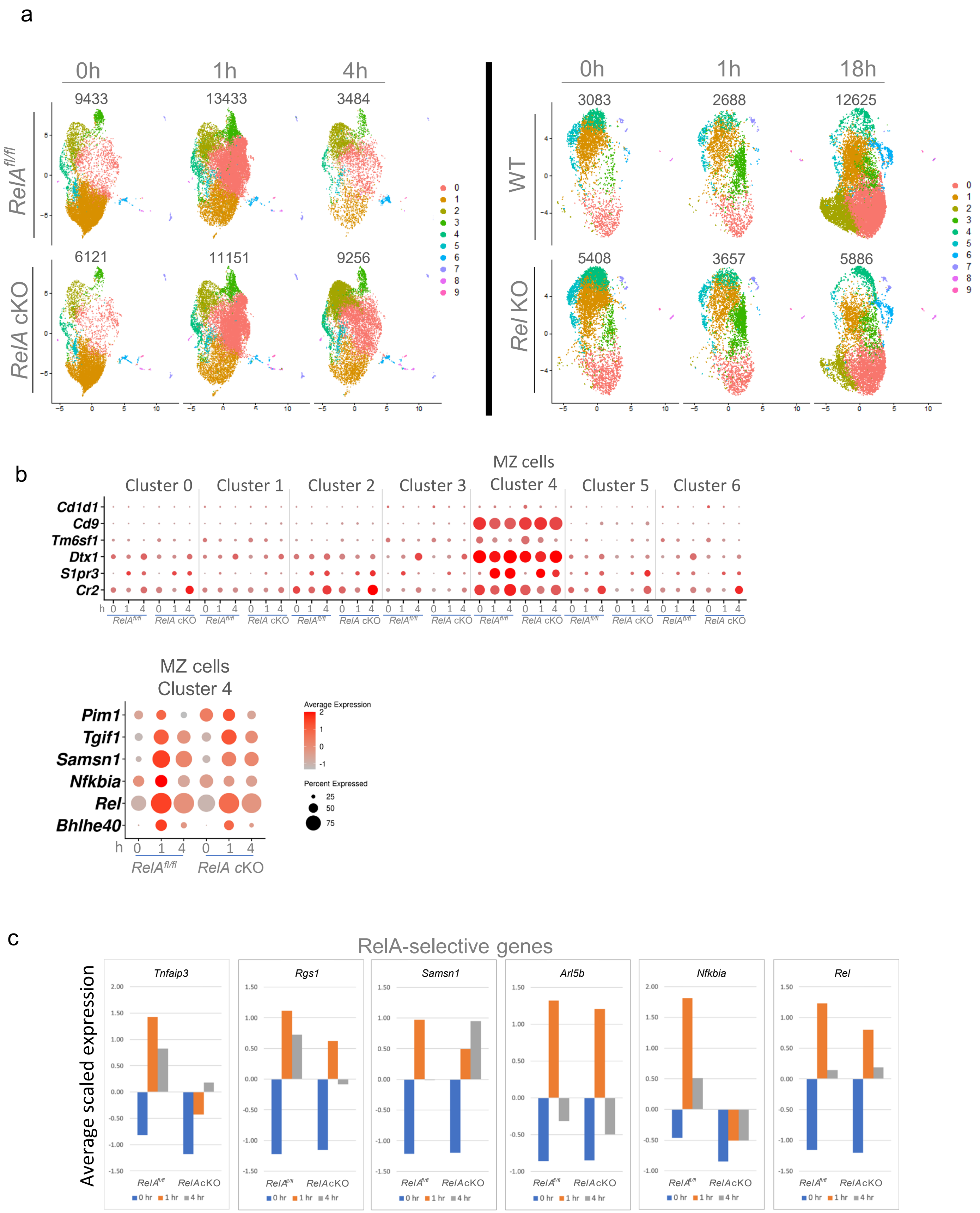

a
0h
1h
4h
9433
13433
3484
6121
11151
9256
RelA fl/fl
RelA cKO
0h
1h
18h
3083
2688
12625
5408
3657
5886
WT
Rel KO
b
MZ cells
Cluster 0
Cluster 1
Cluster 2
Cluster 3
Cluster 4
Cluster 5
Cluster 6
Cd1d1
Cd9
Tm6sf1
Dtx1
S1pr3
Cr2
RelA fl/fl
RelA cKO
MZ cells
Cluster 4
Pim1
Tgif1
Samsn1
Nfkbia
Rel
Bhlhe40
Average Expression
Percent Expressed
c
RelA-selective genes
Average scaled expression
Tnfaip3
Rgs1
Samsn1
Arl5b
Nfkbia
Rel
RelAcKO
0 hr
1 hr
4 hr

Figure S6

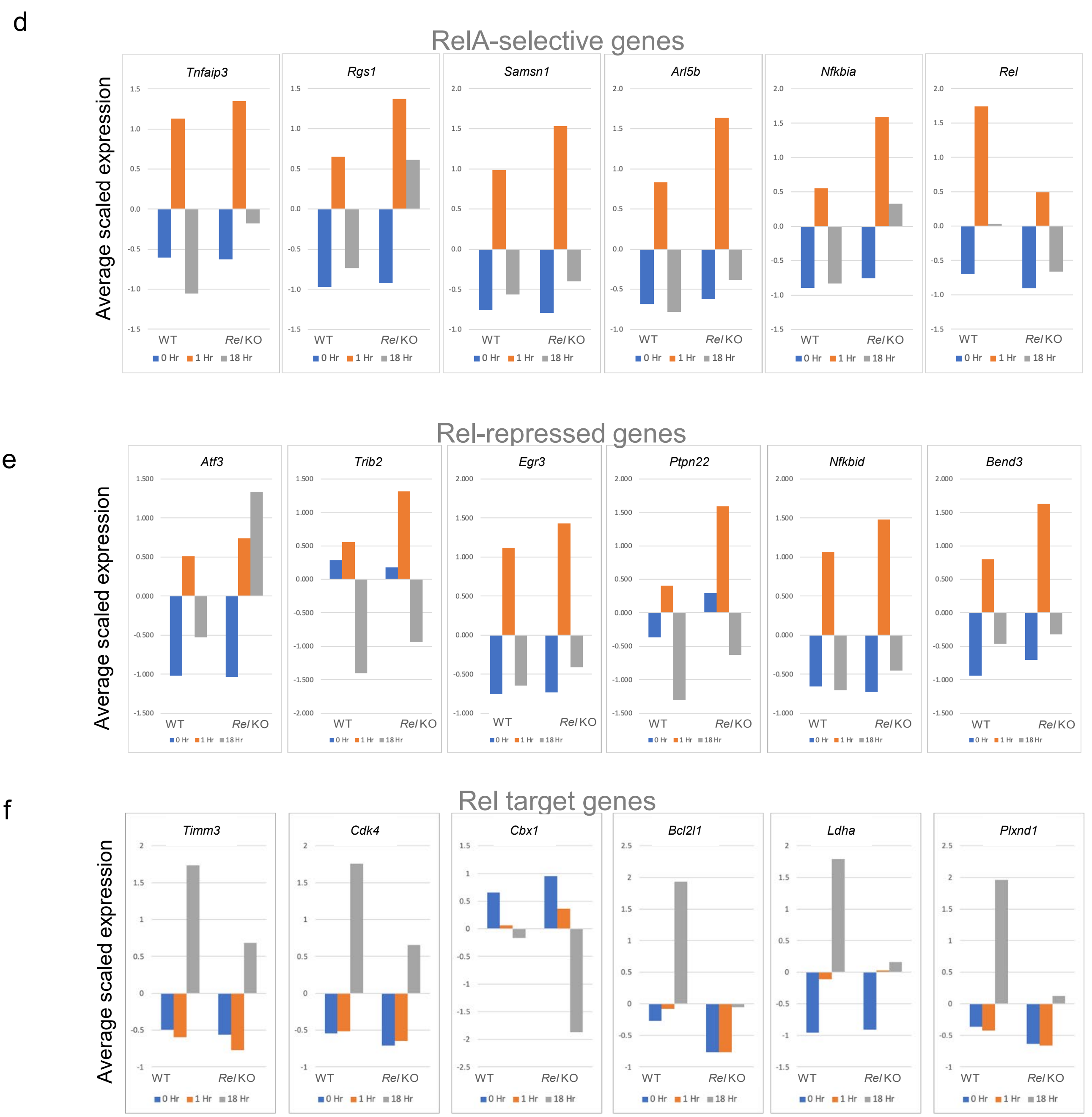

Figure S6: Single cell Seurat analysis of B cell activation in *RelA*$^{fl/fl}$, *RelA* cKO, WT and *Rel* KO cells. **a,** Seurat UMAP visualization of scRNA-Seq libraries generated from *RelA*$^{fl/fl}$ and *RelA* cKO B cells at 0, 1, and 4h (left) and WT and *Rel* KO at 0, 1 and 18h post anti-IgM Fab`2 treatment. The graph-based cluster identities are defined by color in the key to the right of each UMAP set. The number of cells analyzed are indicated. **b,** Average expression and percent of *RelA*$^{fl/fl}$ and *RelA* cKO cells that express marginal zone markers *Cd1d1*, *Cd9*, *Tm6sf1*, *Dtx1*, *S1pr3*, and *Cr2* (top) across clusters 0 - 6 of 11. Cluster 4 exhibits the strongest MZ signature. Expression of RelA-dependent genes *Pim1*, *Tgif1*, *Samsn1*, *Nfkbia*, *Rel*, *Bhlhe40* in cluster 4 (bottom) **c-f,** Scaled expression data is plotted as bar graphs for a subset of genes found in Figure 5 a-d after various induction times. **c,** RelA-selective genes in *RelA*$^{fl/fl}$ and *RelA* cKO B. **d,** Expression of RelA-selective genes in WT and *Rel* KO B. **e,** Rel-repressed genes in WT and *Rel* KO B. **f,** Rel target genes in WT and *Rel* KO B cells.

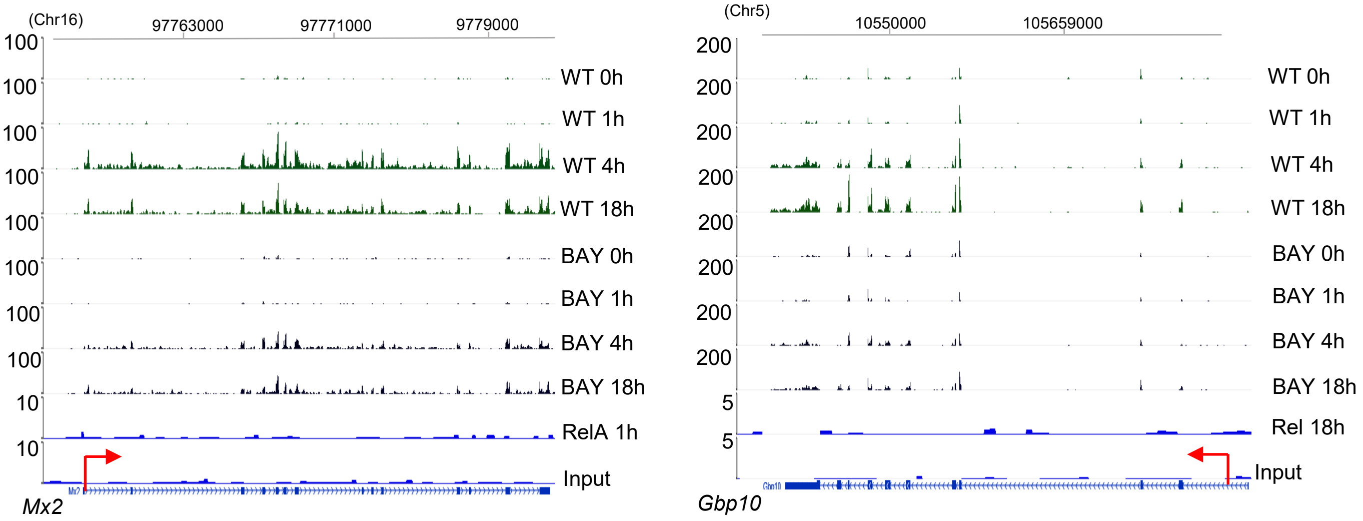

Figure S7: Indirect NF-κB target genes

Mx2 and *Gbp10* are examples of genes that are affected by inhibitor treatment but do not bind either RelA or Rel at any activation time point. The top 8 lines show RNA-Seq tracks at different times after anti-IgM Fab`2 treatment of spleen B cells from C57BL/6 mice in the presence or absence of BAY11-7082 treatment. The bottom 2 lines (including input track) show RelA 1h or Rel 18h ChIP-Seq tracks.

Figure S8

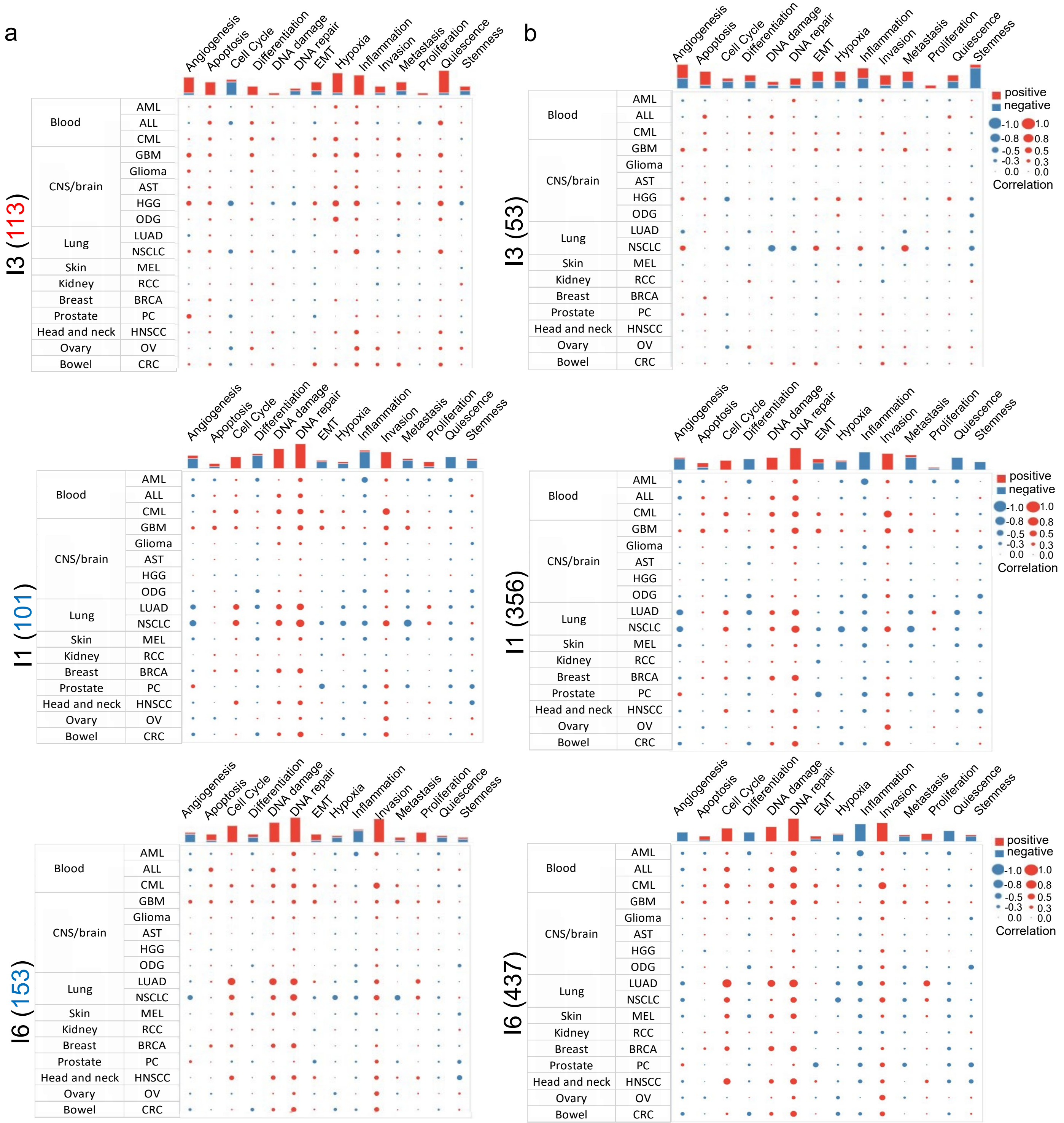

Figure S8: Relationships of direct and indirect NF-κB target genes to cancer.

a, Plots show average correlation of early [I3 (113)] or late [I1 (101) and I6 (153)] direct NF-κB target genes across 14 functional states in 10 cancer types by CancerSEA analysis. The dot size depicts the correlation strength average. The bar graphs illustrate the number of datasets in which the genes are significantly related to the associated function. Blue represents a negative correlation, while red represents a positive correlation. **b,** Relevance of early [I3 (53)] or late [I1 (356) and I6 (437)] indirect NF-κB target genes across 14 functional states in distinct cancers by CancerSEA analysis.

Source data for Figure S3 b

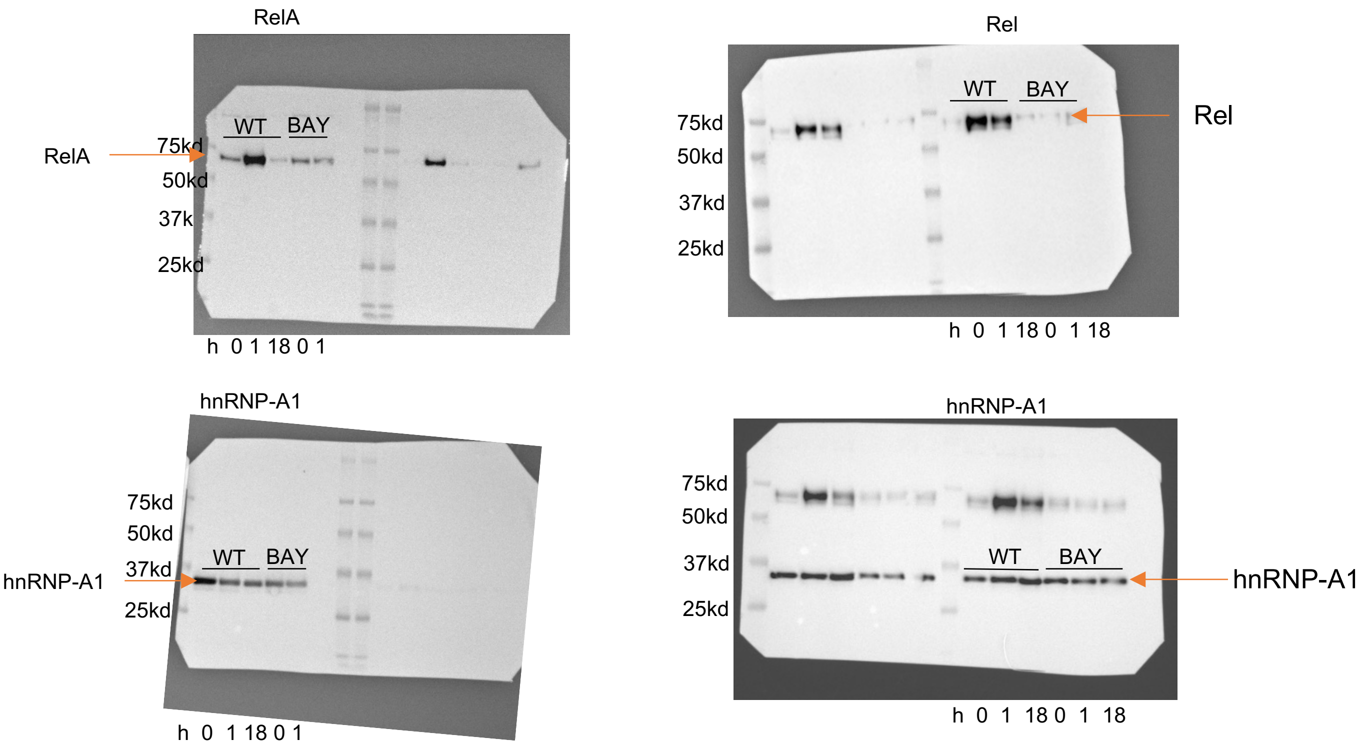

Source data anti-Rel antibody validation

Western Blot on mouse B cell protein extracts for validation of anti-Rel antibody (cat no. sc-71, Santa Cruz Biotechnology), actin was used as a loading control. Lanes — are from other irrelevant mouse strains.

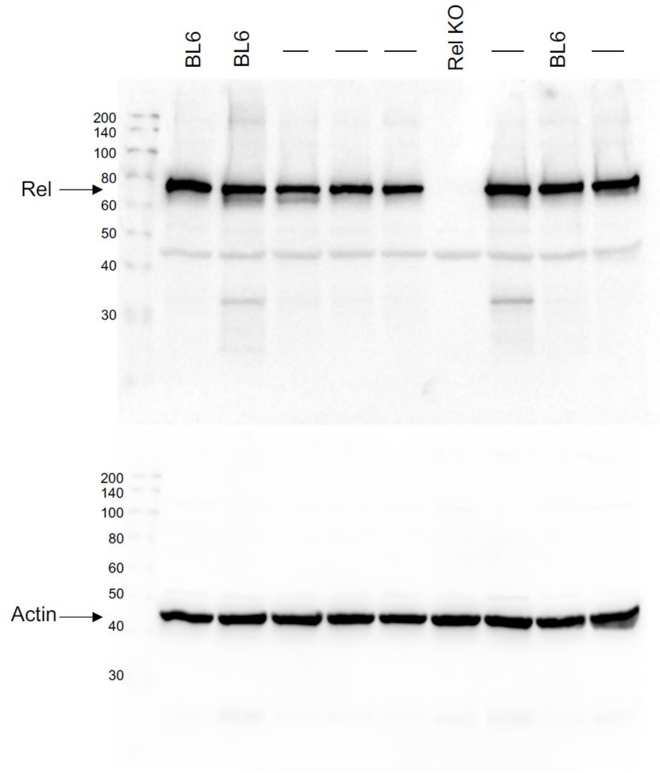

Supplement: Supplementary file 1 — Supplementary Figs. 1–8 with associated legends, full blot image for Supplementary Fig. 3b and full blot image depicting anti-Rel antibody (sc-71) validation. [file 41590_2023_1561_MOESM1_ESM.pdf]
