# Supplementary material for: NF-κB subunits direct kinetically distinct transcriptional cascades in antigen receptor-activated B cells
Source: Nat Immunol. 2023 Jul 31;24(9):1552–64. doi: 10.1038/s41590-023-01561-7 (PMC10457194; doi:10.1038/s41590-023-01561-7)
Supplement: Supplementary file 2 — Reporting Summary [file 41590_2023_1561_MOESM2_ESM.pdf]

nature portfolio

Corresponding author(s): Ranjan Sen

Last updated by author(s): Jun 5, 2023

Reporting Summary

Nature Portfolio wishes to improve the reproducibility of the work that we publish. This form provides structure for consistency and transparency in reporting. For further information on Nature Portfolio policies, see our Editorial Policies and the Editorial Policy Checklist.

Statistics

For all statistical analyses, confirm that the following items are present in the figure legend, table legend, main text, or Methods section.

n/a	Confirmed	
☐	☒	The exact sample size (*n*) for each experimental group/condition, given as a discrete number and unit of measurement
☐	☒	A statement on whether measurements were taken from distinct samples or whether the same sample was measured repeatedly
☐	☒	The statistical test(s) used AND whether they are one- or two-sided<br>*Only common tests should be described solely by name; describe more complex techniques in the Methods section.*
☒	☐	A description of all covariates tested
☒	☐	A description of any assumptions or corrections, such as tests of normality and adjustment for multiple comparisons
☐	☒	A full description of the statistical parameters including central tendency (e.g. means) or other basic estimates (e.g. regression coefficient) AND variation (e.g. standard deviation) or associated estimates of uncertainty (e.g. confidence intervals)
☐	☒	For null hypothesis testing, the test statistic (e.g. *F*, *t*, *r*) with confidence intervals, effect sizes, degrees of freedom and *P* value noted<br>*Give P values as exact values whenever suitable.*
☒	☐	For Bayesian analysis, information on the choice of priors and Markov chain Monte Carlo settings
☒	☐	For hierarchical and complex designs, identification of the appropriate level for tests and full reporting of outcomes
☐	☒	Estimates of effect sizes (e.g. Cohen's *d*, Pearson's *r*), indicating how they were calculated

Our web collection on statistics for biologists contains articles on many of the points above.

Software and code

Policy information about availability of computer code

Data collection	Sequencing data was collected on the HiSeq 2000 (all except the FO or MZ libraries) or the NovaSeq 6000 (FO and MZ libraries). BCL files were demultiplexed and converted to FASTQ files using Illumina's bcl2fastq program (v2.20.0.422). RT-PCR data was collected on the Thermo ABI ViiA 7 Real-Time PCR System using QuantStudio 1.6.1; Image Cytometry was collected on the Amnis ® ImageStream ® X Mk II Imaging Flow Cytometer; Flow cytometry was collected on either the BD Aria Fusion or BD Symphony flow cytometer. Immunoblotting images were collected on the Syngene Imaging System.
Data analysis	The software used for data analysis of ChIP-Seq, ATAC-Seq, bulk RNA-Seq and scRNA-Seq, Image cytometry, flow cytometry and RT-PCR are described in methods. No custom code or algorithms were utilized. ChIP-Seq - Bowtie1 V1.0, Bowtie2 V2.9.2, deepTools V3.4.1, HOMER V4.11 for motif discovery - all with default parameters; ATAC-Seq - FastQC V0.11.8, Trimmomatic V0.33 and Bowtie2 V2.9.2; bulk RNA-Seq - RSEM V1.3.2 , EBSeq V1.24.0, DESeq2 V1.22.0, ShinyGO 0.77 for pathway analysis, MATLAB V9.4 (R2018) for k-means clustering and DiffBind V3.8.4 - all with default parameters; scRNA-Seq - 10X Genomics Cell Ranger suite V.3.0.2 mkfastq, count and combine with standard parameters. Cell Ranger data were processed in R v3.6.3 with Seurat v3.2.3 using default parameters. Flow cytometry was analyzed using FlowJo v10.1 with default parameters. Image Cytometry was analysed using IDEAS 6.0 software (Luminex). GraphPad Prism V9.0 for graphs.

For manuscripts utilizing custom algorithms or software that are central to the research but not yet described in published literature, software must be made available to editors and reviewers. We strongly encourage code deposition in a community repository (e.g. GitHub). See the Nature Portfolio guidelines for submitting code & software for further information.

Data

Policy information about availability of data

All manuscripts must include a data availability statement. This statement should provide the following information, where applicable:

- Accession codes, unique identifiers, or web links for publicly available datasets
- A description of any restrictions on data availability
- For clinical datasets or third party data, please ensure that the statement adheres to our policy

Sequencing datasets generated and analyzed in this publication have been deposited in NCBI's Gene Expression Omnibus (Edgar et al., 2002) and are accessible through GEO Superseries accession number GSE197035 (https://www.ncbi.nlm.nih.gov/geo/query/acc.cgi?acc=GSE197035). ChIP-Seq data is also available at https://wangftp.wustl.edu/~dli/nih-mingming/biowulf/ and may be visualized at WashU Epigenome Browser: https://epigenomegateway.wustl.edu/. TFmotifView results are available here: http://bardet.u-strasbg.fr/tfmotifview/?results=734fKxPAnCh8w1 (RelA 1h); http://bardet.u-strasbg.fr/tfmotifview/?results=H1bHoIdGkHrKPy (Rel 18h).

Field-specific reporting

Please select the one below that is the best fit for your research. If you are not sure, read the appropriate sections before making your selection.

☒ Life sciences ☐ Behavioural & social sciences ☐ Ecological, evolutionary & environmental sciences

For a reference copy of the document with all sections, see nature.com/documents/nr-reporting-summary-flat.pdf

Life sciences study design

All studies must disclose on these points even when the disclosure is negative.

Sample size	ATAC-Seq, ChIP-Seq, bulk RNA-Seq, scRNA-Seq were all carried out with two biological replicates. No statistical methods were used to pre-determine sample sizes but our sample sizes are similar to those reported in previous publications (ATAC-Seq - doi.org/10.1016/j.immuni.2023.03.017; ChIP-Seq - DOI: 10.1016/j.immuni.2018.04.024; bulk RNA-Seq - DOI: 10.1126/science.abn7625; scRNA-Seq - DOI: 10.1016/j.immuni.2021.08.017)
Data exclusions	No data was excluded.
Replication	All the genomic assays were carried out with 2 biological replicates. Pearson correlation coefficients (r) between biological replicates were calculated. For RelA and Rel ChIP-Seq, we only considered peaks that were present in both biological replicates. Differential analysis of RNA-Seq was carried out in DESeq2 using statistics to establish the reproducibility of significant differences. Differential analysis of ATAC-seqcarried out in Diffbind V? using statistics to establish the reproducibility of significant differences was RT-qPCR assays were carried out with at least two independent RNA preparations and graphs provided show data point spread.
Randomization	B cells for each replicate of ChIP-Seq, ATAC-Seq, RNA-Seq, sc RNA-Seq were purified from 5 age-matched mice (mixed male/female). We reasoned that additional randomization was not required for these studies because the data were obtained ex vivo from pooled B cells obtained from multiple mice.
Blinding	Data collection and analysis were not performed blind to the conditions of the experiments.

Reporting for specific materials, systems and methods

We require information from authors about some types of materials, experimental systems and methods used in many studies. Here, indicate whether each material, system or method listed is relevant to your study. If you are not sure if a list item applies to your research, read the appropriate section before selecting a response.

Materials & experimental systems

n/a	Involved in the study	
☐	☒	Antibodies
☒	☐	Eukaryotic cell lines
☒	☐	Palaeontology and archaeology
☐	☒	Animals and other organisms
☒	☐	Human research participants
☒	☐	Clinical data
☒	☐	Dual use research of concern

Methods

n/a	Involved in the study	
☐	☒	ChIP-seq
☐	☒	Flow cytometry
☒	☐	MRI-based neuroimaging

Antibodies

Antibodies used	rabbit-anti mouse RelA (8242S, Cell Signaling Technology); anti-RelA (sc-372, Santa Cruz Biotechnology); anti-Rel (sc-71, Santa Cruz Biotechnology); anti-hnRNP A1 (sc-32301, Santa Cruz Biotechnology); anti-H3K27ac (39133, Active Motif); anti-H3K4me1 (ab8895, Abcam); anti-H3K4me3 (ab8580, Abcam);Horseradish peroxidase coupled goat anti-mouse IgG (sc-2005, Santa Cruz Biotechnology), goat anti-rabbit IgG (sc-2004, Santa Cruz Biotechnology) and goat anti-mouse IgM F(ab`)2 (115-006-075, The Jackson Laboratory); B220+ (103240, BioLegend), AA4.1- (136510, BioLegend), CD21lo (123410, BioLegend) CD23hi(101606, BioLegend); Myc-AF-647 (1:50) (13871, Cell Signaling), Egr2-APC (1:50) (17-6691-82, eBiosciences), Rel-PE (1:200) (12-6111-80, eBiosciences), Ikba-PE (1:100) (7523, Cell Signaling), Dec1-AF-594 (1:100) (1800AF594, Novus Biologicals), Ezh2-PE (1:50) (562478, BD Bioscience), CD72-BV421 (1:200) (740058, BD Bioscience) and HSP90beta (rabbit polyclonal, Invitrogen); anti-rabbit Fab`2-PE antibody (1:1000) (79408, Cell Signaling Technology)
Validation	anti-H3K4me1 (ab8895, Abcam) is specific for mono-methylated Lysine 4 of histone H3 and does not recognize di- or tri-methyl Lysine 4, nor methylation at Lysine 9 per the manufacturer; anti-hnRNP A1 (sc-32301, Santa Cruz Biotechnology) as been utilized in 134 publications; anti-H3K27ac (39133, Active Motif)has been utilized in 77 publications; anti-H3K4me3 (ab8580, Abcam) has been utilized in 1741 publications; goat anti-mouse IgG (sc-2005, Santa Cruz Biotechnology) has been utilized in 929 publications and goat anti-rabbit IgG (sc-2004, Santa Cruz Biotechnology)has been utilized in 347 publications. anti-RelA (sc-372, Santa Cruz Biotechnology) was validated by the manufactor by western blot. anti-Rel (sc-71, Santa Cruz Biotechnology) was validated in our lab by western blot using Rel KO lysates (see source data). The manufacturer of the goat anti-mouse IgM F(ab`)2 (115-006-075, The Jackson Laboratory) antibody has been shown to react with the heavy chain of mouse IgM by ELISA. B220 (103240, BioLegend), RRID AB_11203896; AA4.1 (136510, BioLegend), RRID AB_2275868; CD21 (123410, BioLegend), RRID AB_940413; CD23(101606, BioLegend), RRID AB_312831; Myc-AF-647 (13871, Cell Signaling) has been utilized in 5 publications; Egr2-APC (17-6691-82, eBiosciences), RRID AB_11151502; Rel-PE (12-6111-80, eBiosciences), RRID AB_11042978; Ikba-PE (7523, Cell Signaling) has been utilized in 4 publications; Dec1-AF-594 (1800AF594, Novus Biologicals), RRID:AB_10000524; Ezh2-PE (562478, BD Bioscience), RRID AB_11152951; CD72-BV421 (740058, BD Bioscience), RRID AB_2739823; HSP90beta (PA3-012 rabbit polyclonal, Invitrogen); RRID:AB_2121220; anti-rabbit Fab`2-PE antibody (79408, Cell Signaling Technology) has been utilized in 4 publications.

Animals and other organisms

Policy information about studies involving animals; ARRIVE guidelines recommended for reporting animal research

Laboratory animals	Species - Mus musculus Strains - WT (C57BL/6), RelA cKO (RelAfl/fl x Cd19-cre), RelAfl/fl (Steinbrecher, Harmel-Laws et al. 2008), Cd19-cre (006785, The Jackson Laboratory) and Rel KO (Rel-/-)(Cheng, S., Hsia C.Y. et al. 2003); Age - 8-12 weeks; Sex - both sexes were used for experiments.
Wild animals	No wild animals were used.
Field-collected samples	No field-collected samples were used.
Ethics oversight	Institutional Animal Welfare Assurance Number – NIH Intramural Research Program – D16-00602; NIA AAALAC Unit Number – 000401.

Note that full information on the approval of the study protocol must also be provided in the manuscript.

ChIP-seq

Data deposition

☒ Confirm that both raw and final processed data have been deposited in a public database such as GEO.

☒ Confirm that you have deposited or provided access to graph files (e.g. BED files) for the called peaks.

Data access links<br>*May remain private before publication.*	GEO accession number specific to ChIP-Seq is GSE197032.
Files in database submission	RelA 0h1.fastq.gz, RelA 0h2.fastq.gz, RelA 1h1.fastq.gz, RelA 1h2.fastq.gz, RelA 4h1.fastq.gz, RelA 4h2.fastq.gz, Rel 0h1.fastq.gz, Rel 0h2.fastq.gz, Rel 1h1.fastq.gz, Rel 1h2.fastq.gz, Rel 18h1.fastq.gz, Rel 18h2.fastq.gz, H3K27ac 0h1.fastq.gz, H3K27ac 0h2.fastq.gz, H3K4me1 0h1.fastq.gz, H3K4me1 0h2.fastq.gz, H3K4me3 0h1.fastq.gz, H3K4me3 0h2.fastq.gz, H3K4me3 1h1.fastq.gz, H3K4me3 1h2.fastq.gz, H3K4me3 18h1.fastq.gz,H3K4me3 18h2.fastq.gz, RelA 0h1.bw, RelA 0h2.bw, RelA 1h1.bw, RelA 1h2.bw, RelA 4h1.bw, RelA 4h2.bw, Rel 0h1.bw, Rel 0h2.bw, Rel 1h1.bw, Rel 1h2.bw, Rel 18h1.bw, Rel 18h2.bw, H3K27ac 0h1.bw, H3K27ac 0h2.bw, H3K4me1 0h1.bw, H3K4me1 0h2.bw, H3K4me3 0h1.bw, H3K4me3 0h2.bw, H3K4me3 1h1.bw, H3K4me3 1h2.bw, H3K4me3 18h1.bw, H3K4me3 18h2.bw
Genome browser session<br>(e.g. UCSC)	WashU Epigenome Browser: https://epigenomegateway.wustl.edu/ ; data: https://wangftp.wustl.edu/~dli/nih-mingming/biowulf/

Methodology

Replicates	2 replicates for each ChIP-seq sample
Sequencing depth	RelA ChIP-seq reads range: 18 million - 27 million; Rel ChIP-seq reads range: 10 million - 32 million; H3K27ac ChIP-seq reads range: 36

Sequencing depth	million - 38 million; H3K4me3 ChIP-seq reads range: 17 million - 29 million; H3K4me1 ChIP-seq reads range: 36 million - 44 million. Total reads information for all samples are shown in Figure S1d and Figure S2a.
Antibodies	anti-RelA (sc-372, Santa Cruz Biotechnology); anti-Rel (sc-71, Santa Cruz Biotechnology); anti-H3K27ac (39133, Active Motif); anti-H3K4me1 (ab8895, Abcam); anti-H3K4me3 (ab8580, Abcam)
Peak calling parameters	CisGenome software (http://www.biostat.jhsph.edu/~hji/cisgenome/) was used for peak calling with a window size of 200 bases and a threshold of 4 reads (2 from each strand), FDR<0.1 and fold change >2. Peaks separated by less than 200 bases were merged into one peak.
Data quality	Quality check for both RelA and Rel ChIP-seq, was performed by 1) calculating Pearson correlation coefficient (r) between replicates and 2) comparing peak call numbers. RelA 0h replicates r=0.7; RelA1h replicates r=0.81; RelA 4h replicates r=0.77; Rel 0h replicates r=0.92;Rel 1h replicates r=0.93; Rel 18h replicates r=0.97; RelA 0h1 peak number=12484;RelA 0h2 peak number=5116; RelA 1h1 peak number=57105;RelA 1h2 peak number=39934; RelA 4h1 peak number=14461;RelA 4h2 peak number=21015; Rel 0h1 peak number=1353;Rel 0h2 peak number=780;Rel 1h1 peak number=2827;Rel 1h2 peak number=2548; Rel 18h1 peak number=15163; Rel 18h2 peak number=24410.<br>For all other histone ChIP-seq we only did replicates pearson correlation, peak calling are not done on histone ChIP-seq, instead we use deepTools to see the heapmaps results of some specific regions. H3K4me3 0h replicates Pearson=0.98;H3K4me3 1h replicates Pearson=0.95;H3K4me3 18h replicates Pearson=0.99;H3K27ac 0h replicates Pearson=0.97; H3K4me1 0h replicates Pearson=0.93.
Software	Bowtie1 V1.0 software was used to map quality-filtered reads from demultiplexed FASTQ files to mouse genome assembly mm9 (Ensembl v67) with default options. CisGenome software (http://www.biostat.jhsph.edu/~hji/cisgenome/) was used for peak calling with a window size of 200 bases and a threshold of 4 reads (2 from each strand), FDR<0.1 and fold change >2.<br>HOMER annotatePeaks.pl was used to annotate peaks with default parameters (promoter regions were defined from −1 kb to +100 bp). The programs findMotifs-Genome.pl and findMotifs.pl were used to identify transcription factor binding motifs within peaks or promoter regions . The computeMatrix and plotHeatmap modules of the deepTools package were used to produce heat map.

Flow Cytometry

Plots

Confirm that:

☒ The axis labels state the marker and fluorochrome used (e.g. CD4-FITC).

☒ The axis scales are clearly visible. Include numbers along axes only for bottom left plot of group (a 'group' is an analysis of identical markers).

☒ All plots are contour plots with outliers or pseudocolor plots.

☒ A numerical value for number of cells or percentage (with statistics) is provided.

Methodology

Sample preparation	Isolated B cells (0.5x106) from spleens were fixed with 2% paraformaldehyde (15713, Electron Microscopy) for 10 mins in the dark at room temperature. Fixed cells were washed with PBS and resuspended in ice cold Perm Buffer III (558050, BD Biosciences) and incubated overnight at -20°C. Cells were washed with PBS and resuspended in BD perm/wash buffer (554723, BD Biosciences).
Instrument	BD FACS-Aria Fusion; BD Symphony
Software	FlowJo Version 10.1
Cell population abundance	Splenic naïve B cells were comprised of 74.1 % follicular (FO) and 9.04 % marginal (MZ) cells
Gating strategy	Gating shown in the Supplementary figures S3c, S3g, and S4e

☒ Tick this box to confirm that a figure exemplifying the gating strategy is provided in the Supplementary Information.
