# Supplementary material for: NF-κB subunits direct kinetically distinct transcriptional cascades in antigen receptor-activated B cells
Source: Nat Immunol. 2023 Jul 31;24(9):1552–64. doi: 10.1038/s41590-023-01561-7 (PMC10457194; doi:10.1038/s41590-023-01561-7)
Supplement: Supplementary file 4 — ChIP–PCR primers used in Fig. 1c and Supplementary Figs. 1c and 4d. [file 41590_2023_1561_MOESM4_ESM.pdf]

ChIP-PCR primers

Main Figures

Figure 1c

Primer Name	Sequence
Bach2-F	GCCACTTACTTGGGGAAATG
Bach2-R	GACAACTGCCAACGTCTTGA
Bcl2l1-F	GTTGCCCACACAAAGTCAAG
Bcl2l1-R	TTGCCATCATAGGGTTTTCC
Ccl3-F	AGGTCAGGTGCTCTCTCAGC
Ccl3-R	GAGCCCTCGAAGTGTGACTC
Cdc274-F	CTCTTCCTCATTCCCCTTCA
Cdc274-R	CTGCGTGGGTCTGGAGTTA
Dennd4a-F	CCTTTTCCCTGACCTCTGTCT
Dennd4a-R	TCCTCTTTCACAAGCATCCA
Etv6-F	TGTTACTTCTGTCCCCACCA
Etv6-R	TCCACTTCCACTTCCTCCTT
Gm14005-F	CAGGCACAATGACATCTGCT
Gm14005-R	CTCTGACTCAACGGGGAAAC
Gm20098-F	GTGACTTCCGCTCTGTAGCA
Gm20098-R	GGCAACCTATCTCTGTGTGG
Gpr132-F	GAACTCCCCTAACCACAACG
Gpr132-R	CCTGAGAAGAGCCATCATCC
Klf10-F	AGACGGGAGCACTGAGACA
Klf10-R	ACACCTTTGCCGCTGATT
Mcl1-F	GGAAAGGGTGGGATGTCA
Mcl1-R	TGCCTGAGAAGAAAAGCAAG
Mcm9-F1	TGGAATCGCCTCAAAGAAC
Mcm9-R1	TGCTTCAACCCTCCCTTG
Nfkbia distal- F	AACTGGCTCGTCCTCCACT
Nfkbia distal-R	CACTCAGGGCTCATCAAAAAG
Pdcd1lg2-F	ACGACTGCTTGGGGAAGTT
Pdcd1lg2-R	CACCTGGGGATGACACTCTAA
Psma6-F	TGTGGGCTTTGCTAAACAGA
Psma6-R	TGCCTCGATGGAAAGTTTG
Slamf6-F	CAGACACACAACTGGCACTACA
Slamf6-R	AACCGAAGGTGGAACATCC
Stim2-F1	TGGCTTCTTGGGTTGGTAAT
Stim2-R1	CCTCTCTCCTTGGTCCTTTG
Tnfaip3-F	TGATGGGAACTGGAAATGC
Tnfaip3-R	CTGTGTGTCTGGGTGGTTTTT
Tpd52-F	ATGACCCAGAGGAAATGGAA
Tpd52-R	CAGCCAGTGAGCCTAATCAA
Zfp619-F	GGTTTACGAGGATTCAACAAGG
Zfp619-R	AGACCTGGGAGGGTTTCAGT

Supplementary Figures

Figure S1c

Primer Name	Sequence
Bcl2l1 F	CCGAGACGCAAAAGGAGT
Bcl2l1 R	GGGACAGATGGATGAAGGAA
Nfkbia-F	CTGAGTGGCTGGAAAGTCCT
Nfkbia-R	CTGGCAAATCGCTAAGAGG
Negative-control-F	GCCATCTTTCTACCTTCCACA
Negative-control-R	CTCCTGTCTGCCCCTGTTAT

Figure S4e

Primer Name	Sequence
Arid3a-F	GGGGTTTTTAAGCCCATTTC
Arid3a-R	GCAAGATGGATAAGCCAAGC
Arid3a-neg-F	TAGACGGGGAATCTGGACAC
Arid3a_neg_R	GTCGGGTCAGCAACAAAGA
Bcl11a-F	CATTTGGTGATTCTAAGGCTGA
Bcl11a-R	GGTTTGAGGAGCAGGAAGAC
Bcl11a-neg-F	GTTGCTGGAGGTGGGTGTA
Bcl11a-neg-R	GCAGTCCTATGGTGAGAGTTGA
Bcl6b-F	AGTGGGAGAAGGAGGAGTGA
Bcl6b-R	GGACAATGGAAGGAAACGAA
Bcl6b_neg_F	GGACAGGAGGAAAGGGCTAT
Bcl6b_neg_R	CCCACACAGCAACACTCATT
Cbx3-F	GAGGAAGGAGACGGGAAAAT
Cbx3-R	TAGGGGCTCGGTTGTGTT
Cbx3-neg-F	ACAGTCTTTGTGGCAGCAGA
Cbx3_neg_R	TTCTCACCCCCTCCCTTT
Hhex-F	AAGCGCAGTGGAAAAGTTG
Hhex-R	AAGGTGCAGGAAAAGCAATC
Hhex-neg-F	ACCTCAACAGGCGATGACTT
Hhex_neg_R	CCAGCCACAAAGATGGAAAT
Ldha-F	AAGAGTCGCAGTCCTTCCTC
Ldha-R	TCTACTTCACGCAAACCTCAA
Ldha_neg_F	TTGCGTCAGGGAAATGTATG
Ldha_neg_R	AAGGTCGTGGGAAAGATGG
Mki67-F	AAAACGGCGGTTCAAATTC
Mki67-R	CCTCTGGACTTCCCCAATC
Mki67-neg-F	AGAGAGACACACTCAGGATGGA
Mki67-neg-R	TGTTGGGGAAGGATTAGGAG
Nfkbie-F	ATGATGGCTTGTGTGGAATG
Nfkbie-R	AACAGGGAGGACAGGAGGA
Nfkbie_neg_F	CTCAGCCTCAACTCCTCTCC
Nfkbie_neg_R	TCTCTTCTCCCCAGCCTTC
Ppp1ca-F	GAGAAGTCAGCGATTAACATGC
Ppp1ca-R	GCCGCGTTCAGATGAGTTA
Ppp1ca-neg-F	TCTGAGGAGGGCTTGAGGT
Ppp1ca_neg_R	CAAAAGGGTTCCAGTTCCAC
Xrcc-1-F	CACGTAGGACCCAATCACTG
Xrcc-1-R	CTCAGGTTCCACCACTCTCC
Xrcc1_neg_F	ATTTCAACCCTCCCCTGATT
Xrcc1_neg_R	GAGCGAGTCCAGCACAAGTA
