# Supplementary material for: NF-κB subunits direct kinetically distinct transcriptional cascades in antigen receptor-activated B cells
Source: Nat Immunol. 2023 Jul 31;24(9):1552–64. doi: 10.1038/s41590-023-01561-7 (PMC10457194; doi:10.1038/s41590-023-01561-7)
Supplement: Supplementary file 8 — Supporting data for Supplementary Fig. 4c. Gene expression analysis of RelA-selective genes in ex vivo RelA deleted B cells (by qRT–PCR CT values provided). [file 41590_2023_1561_MOESM8_ESM.pdf]

Fig S4c: Gene expression of RelA selective genes in ex vivo RelA deleted B cells

Experiment 1

	Target name	WT 0h	WT 1h	WT 4h	WT 18h	WT+TAT 0h	WT+TAT 1h	WT+TAT 4h	WT+TAT 18h
Replicate 1	Bactin	19.13	19.16	18.74	18.32	19.50	19.42	18.26	17.62
Replicate 2	Bactin	19.14	18.76	18.63	18.23	19.41	19.46	18.39	17.67
Replicate 1	Nfkbia	20.15	18.73	20.06	21.17	20.13	18.94	19.73	20.31
Replicate 2	Nfkbia	20.14	18.90	20.09	21.19	19.66	18.93	19.77	20.54
Replicate 1	Tnfaip3	22.77	21.44	23.14	24.80	24.11	22.23	22.87	23.52
Replicate 2	Tnfaip3	22.89	21.39	23.07	24.86	24.13	22.12	22.87	23.65
Replicate 1	Gadd45b	23.42	22.01	23.50	25.83	24.01	22.33	24.21	25.35
Replicate 2	Gadd45b	23.65	21.80	23.75	26.19	24.01	22.16	24.20	25.20

	Target name	WT 0h	WT 1h	WT 4h	WT 18h	WT+TAT 0h	WT+TAT 1h	WT+TAT 4h	WT+TAT 18h
Replicate 1	Bactin	19.24	18.78	18.95	19.16	19.63	19.09	18.94	18.67
Replicate 2	Bactin	19.14	18.93	19.27	19.43	19.52	19.10	18.82	18.74
Replicate 1	Pim-1	24.06	22.04	24.83	26.78	25.24	22.45	24.43	26.10
Replicate 2	Pim-1	23.95	21.85	24.95	26.25	24.88	22.33	24.24	25.44
Replicate 1	Ripk2	24.82	23.18	25.32	25.59	25.12	23.35	24.94	24.88
Replicate 2	Ripk2	24.74	23.34	25.29	25.76	24.78	23.27	24.66	24.24
Replicate 1	Ing3	23.60	23.11	24.06	24.34	23.97	23.12	23.59	23.77
Replicate 2	Ing3	23.46	22.74	23.72	24.27	23.65	22.97	23.30	23.16
Replicate 1	Tgif1	21.77	20.28	21.98	23.54	21.71	19.96	21.15	22.04
Replicate 2	Tgif1	22.32	20.35	21.93	23.05	21.65	20.35	21.15	22.54
Replicate 1	Samsn1	22.56	21.43	22.40	22.92	22.87	21.47	22.10	22.22
Replicate 2	Samsn1	22.61	21.39	22.32	22.94	22.76	21.42	21.99	21.60

	Target name	WT 0h	WT 1h	WT 4h	WT 18h	WT+TAT 0h	WT+TAT 1h	WT+TAT 4h	WT+TAT 18h
Replicate 1	Bactin	19.42	19.56	19.89	19.67	20.25	19.42	18.54	18.88
Replicate 2	Bactin	19.29	19.58	19.52	19.68	19.94	19.52	19.29	19.01
Replicate 1	Bhlhe40	20.55	19.94	21.04	22.43	20.79	19.69	20.10	21.45

Replicate 2	Bhlhe40	21.31	19.96	21.09	22.44	21.22	19.73	20.37	21.46

	Target name	RelA fl/fl 0h	RelA fl/fl 1h	RelA fl/fl 4h	RelA fl/fl 18h	RelA fl/fl + TAT 0h	RelA fl/fl + TAT 1h	RelA fl/fl + TAT 4h	RelA fl/fl + TAT 18h
Replicate 1	B actin	19.60	18.69	18.48	18.36	19.31	18.60	19.49	17.03
Replicate 2	B actin	19.72	18.46	18.44	18.22	19.39	19.72	19.31	16.90
Replicate 1	Nfkbia	19.76	18.19	20.01	21.36	20.13	19.61	20.72	20.55
Replicate 2	Nfkbia	19.73	17.96	20.02	21.39	20.13	20.34	20.87	20.54
Replicate 1	Tnfaip3	23.08	21.16	23.36	24.81	24.75	23.40	23.50	23.19
Replicate 2	Tnfaip3	23.23	21.09	23.34	24.98	24.67	23.77	23.74	23.63
Replicate 1	Gadd45b	23.93	21.17	24.07	26.16	24.27	22.73	25.10	24.96
Replicate 2	Gadd45b	24.10	21.12	24.00	26.01	24.36	22.91	25.13	24.83

	Target name	RelA fl/fl 0h	RelA fl/fl 1h	RelA fl/fl 4h	RelA fl/fl 18h	RelA fl/fl + TAT 0h	RelA fl/fl + TAT 1h	RelA fl/fl + TAT 4h	RelA fl/fl + TAT 18h
Replicate 1	Bactin	18.25	17.88	17.64	17.64	19.09	17.83	17.84	20.47
Replicate 2	Bactin	18.15	18.29	17.76	17.53	19.44	17.95	17.99	20.51
Replicate 1	Pim1	24.45	21.25	22.59	24.78	25.66	21.64	22.88	28.17
Replicate 2	Pim1	24.22	21.68	22.63	24.96	25.69	21.66	22.90	28.34
Replicate 1	Ing3	22.98	21.87	22.99	23.40	23.94	22.14	23.96	27.37
Replicate 2	Ing3	23.22	21.68	22.94	23.81	23.60	22.21	23.81	27.11
Replicate 1	Bhlhe40	20.70	19.09	19.89	21.58	21.63	19.47	20.66	25.24
Replicate 2	Bhlhe40	20.45	18.51	19.89	22.14	21.47	19.21	19.97	24.74

	Target name	RelA fl/fl 0h	RelA fl/fl 1h	RelA fl/fl 4h	RelA fl/fl 18h	RelA fl/fl + TAT 0h	RelA fl/fl + TAT 1h	RelA fl/fl + TAT 4h	RelA fl/fl + TAT 18h
Replicate 1	Bactin	19.70	19.80	19.68	19.55	19.56	18.98	19.12	17.45
Replicate 2	Bactin	19.84	19.37	19.62	19.57	18.87	18.80	19.31	18.29
Replicate 1	Ripk2	23.88	22.05	24.99	29.87	25.18	23.43	26.24	25.61
Replicate 2	Ripk2	23.88	22.61	25.02	29.72	25.18	23.43	25.98	24.74

	Target name	RelA fl/fl 0h	RelA fl/fl 1h	RelA fl/fl 4h	RelA fl/fl 18h	RelA fl/fl + TAT 0h	RelA fl/fl + TAT 1h	RelA fl/fl + TAT 4h	RelA fl/fl + TAT
Replicate 1	Bactin	18.01	17.97	17.63	17.78	18.75	17.87	17.89	20.77
Replicate 2	Bactin	17.99	17.87	17.74	18.02	18.77	17.82	18.04	20.94
Replicate 1	Tgif1	20.37	19.19	19.77	21.99	21.07	19.15	20.27	25.07
Replicate 2	Tgif1	20.34	19.07	19.90	22.35	21.25	19.28	20.19	24.86
Replicate 1	Samsn1	22.19	19.99	20.41	21.55	22.89	20.17	21.15	24.37
Replicate 2	Samsn1	22.40	19.91	20.58	21.59	22.89	20.14	21.27	24.42

Experiment 2

	Target name	WT 0h	WT 1h	WT 4h	WT 18h	WT+TAT 0h	WT+TAT 1h	WT+TAT 4h	WT+TAT 18h
Replicate 1	Bactin	19.13	18.26	16.97	16.05	18.48	18.58	17.10	15.81
Replicate 2	Bactin	19.14	18.60	16.52	16.30	18.29	18.66	17.34	15.67
Replicate 1	Nfkbia	20.33	17.93	18.36	18.52	19.32	18.27	19.04	18.78
Replicate 2	Nfkbia	20.49	17.95	18.51	18.52	19.40	18.26	19.05	18.72
Replicate 1	Tnfaip3	23.43	21.16	21.29	22.11	23.36	21.57	21.05	22.43
Replicate 2	Tnfaip3	23.63	21.41	21.49	22.00	23.66	21.68	21.30	22.37
Replicate 1	Gadd45b	23.54	20.98	21.40	22.99	22.97	21.08	22.04	23.82
Replicate 2	Gadd45b	24.08	20.87	21.72	23.14	23.34	20.93	22.52	23.93

	Target name	WT 0h	WT 1h	WT 4h	WT 18h	WT+TAT 0h	WT+TAT 1h	WT+TAT 4h	WT+TAT 18h
Replicate 1	Bactin	19.12	17.97	17.44	17.60	18.29	17.82	17.59	17.51
Replicate 2	Bactin	18.99	17.97	17.27	17.41	17.94	17.70	17.62	18.09
Replicate 1	Pim1	24.31	21.36	22.64	25.08	25.09	21.17	23.06	26.24
Replicate 2	Pim1	24.58	21.34	22.47	25.06	24.77	21.10	23.40	26.56
Replicate 1	Ripk2	24.86	22.30	23.71	24.86	24.49	22.06	23.94	25.13
Replicate 2	Ripk2	24.79	22.23	24.37	24.91	23.96	23.08	23.79	24.41
Replicate 1	Ing3	24.59	22.17	22.90	24.11	23.35	21.92	23.33	24.68
Replicate 2	Ing3	23.74	22.15	23.31	24.48	24.78	22.13	23.65	24.49

	Target name	WT 0h	WT 1h	WT 4h	WT 18h	WT+TAT 0h	WT+TAT 1h	WT+TAT 4h	WT+TAT 18h
Replicate 1	Bactin	18.89	17.84	17.32	18.07	18.08	17.60	17.72	17.54

Replicate 2	Bactin	19.56	18.19	17.37	18.11	17.97	17.69	17.94	17.77
Replicate 1	Tgif1	21.19	19.32	19.54	**22.00**	20.65	19.00	**19.94**	22.10
Replicate 2	Tgif1	22.04	19.25	19.67	**22.33**	20.76	19.00	**20.07**	22.08
Replicate 1	Samsn1	23.10	20.23	20.24	21.85	22.62	19.85	20.54	**21.33**
Replicate 2	Samsn1	24.45	20.57	20.61	21.82	22.44	19.93	20.73	**21.71**

	**Target name**	**WT 0h**	**WT 1h**	**WT 4h**	**WT 18h**	**WT+TAT 0h**	**WT+TAT 1h**	**WT+TAT 4h**	**WT+TAT 18h**
Replicate 1	B actin	19.50	17.85	17.48	16.99	18.01	17.66	17.66	16.54
Replicate 2	B actin	19.52	17.97	17.43	17.30	18.11	17.89	17.62	16.66
Replicate 1	Bhlhe40	21.81	18.93	18.81	20.03	19.97	18.58	18.96	20.05
Replicate 2	Bhlhe40	21.61	18.93	18.90	20.12	19.72	18.58	18.93	19.94

	**Target name**	**RelA fl/fl 0h**	**RelA fl/fl 1h**	**RelA fl/fl 4h**	**RelA fl/fl 18h**	**RelA fl/fl + TAT 0h**	**RelA fl/fl + TAT 1h**	**RelA fl/fl + TAT 4h**	**RelA fl/fl + TAT 18h**
Replicate 1	Bactin	18.46	18.57	17.24	16.38	17.97	18.13	17.17	17.71
Replicate 2	Bactin	18.36	18.64	18.11	15.93	19.21	18.44	16.47	18.90
Replicate 1	Nfkbia	19.59	17.98	18.56	18.35	19.37	19.26	19.32	21.91
Replicate 2	Nfkbia	19.68	17.95	18.61	18.37	21.96	19.30	18.70	22.30
Replicate 1	Tnfaip3	22.95	21.53	21.53	22.10	23.87	22.71	21.12	25.14
Replicate 2	Tnfaip3	23.16	21.51	21.82	22.08	24.82	22.55	23.93	25.07
Replicate 1	Gadd45b	23.38	21.27	22.97	22.97	22.78	21.98	21.31	25.84
Replicate 2	Gadd45b	23.87	21.27	22.96	23.28	26.03	22.17	24.62	26.35

	**Target name**	**RelA fl/fl 0h**	**RelA fl/fl 1h**	**RelA fl/fl 4h**	**RelA fl/fl 18h**	**RelA fl/fl + TAT 0h**	**RelA fl/fl + TAT 1h**	**RelA fl/fl + TAT 4h**	**RelA fl/fl + TAT 18h**
Replicate 1	Bactin	19.16	18.81	18.63	18.54	18.85	18.83	17.93	17.89
Replicate 2	Bactin	18.94	18.23	19.48	18.67	19.08	19.08	18.27	17.92
Replicate 1	Pim1	23.82	22.62	25.04	27.45	25.05	22.96	23.19	26.00
Replicate 2	Pim1	23.75	22.02	24.72	25.99	25.69	23.11	23.97	25.98

	**Target name**	**RelA fl/fl 0h**	**RelA fl/fl 1h**	**RelA fl/fl 4h**	**RelA fl/fl 18h**	**RelA fl/fl + TAT 0h**	**RelA fl/fl + TAT 1h**	**RelA fl/fl + TAT 4h**	**+ TAT 18h**

Replicate 1	Bactin	19.56	21.13	20.43	21.14	20.03	20.31	21.93	19.36
Replicate 2	Bactin	20.03	21.09	20.37	21.65	20.48	20.32	21.90	19.61
Replicate 1	Ing3	24.42	24.89	25.49	26.84	25.75	24.93	26.22	25.62
Replicate 2	Ing3	24.44	24.58	25.31	27.68	26.08	24.95	26.25	25.71

	**Target name**	**RelA fl/fl 0h**	**RelA fl/fl 1h**	**RelA fl/fl 4h**	**RelA fl/fl 18h**	**RelA fl/fl + TAT 0h**	**RelA fl/fl + TAT 1h**	**RelA fl/fl + TAT 4h**	**RelA fl/fl + TAT 18h**
Replicate 1	Bactin	21.08	20.31	19.48	18.30	21.11	19.94	19.97	21.36
Replicate 2	Bactin	20.78	20.28	19.52	18.28	22.09	19.94	20.00	21.48
Replicate 1	Ripk2	25.95	23.16	24.93	24.33	27.43	23.88	25.07	27.37
Replicate 2	Ripk2	25.91	22.97	25.10	24.43	26.87	24.40	25.07	27.62

	**Target name**	**RelA fl/fl 0h**	**RelA fl/fl 1h**	**RelA fl/fl 4h**	**RelA fl/fl 18h**	**RelA fl/fl + TAT 0h**	**RelA fl/fl + TAT 1h**	**RelA fl/fl + TAT 4h**	**RelA fl/fl + TAT 18h**
Replicate 1	Bactin	19.76	19.19	18.77	18.34	19.19	18.97	17.77	17.77
Replicate 2	Bactin	19.73	19.01	18.79	18.39	19.10	18.90	18.10	17.57
Replicate 1	Tgif1	21.98	20.39	21.24	22.89	20.80	20.21	20.07	21.99
Replicate 2	Tgif1	21.93	19.38	21.26	23.53	21.13	19.76	20.61	22.42
Replicate 1	Samsn1	22.99	20.73	22.10	23.25	22.31	21.02	21.89	21.86
Replicate 2	Samsn1	22.91	20.61	21.98	23.23	22.26	21.10	21.79	22.18
Replicate 1	Bhlhe40	21.04	19.83	20.28	21.64	20.21	20.10	19.35	20.78
Replicate 2	Bhlhe40	20.77	19.55	20.15	21.37	20.23	19.82	19.32	20.62
