# Supplementary material for: NF-κB subunits direct kinetically distinct transcriptional cascades in antigen receptor-activated B cells
Source: Nat Immunol. 2023 Jul 31;24(9):1552–64. doi: 10.1038/s41590-023-01561-7 (PMC10457194; doi:10.1038/s41590-023-01561-7)
Supplement: Supplementary file 9 — Supporting data for Supplementary Fig. 4e. ChIP–PCR analysis of Rel target genes at 18 h after anti-IgM treatment (CT values provided). [file 41590_2023_1561_MOESM9_ESM.pdf]

Figure S4e: ChIP-PCR analysis of Rel target genes at 18h after anti-IgM treatment

Experiment 1

	Target Name	Input 18h-neg	Rel 18h-neg	Target Name	Input-target	Rel 18h-target
Replicate 1	Ppp1ca -ve	28.68	31.04	Ppp1ca	29.19	29.98
Replicate 2	Ppp1ca -ve	28.68	29.14	Ppp1ca	30.43	29.53
Replicate 1	Nfkbie -ve	28.91	29.06	Nfkbie	28.68	27.40
Replicate 2	Nfkbie -ve	27.86	29.20	Nfkbie	28.99	26.97
Replicate 1	Ldha -ve	28.90	30.95	Ldha	30.22	29.77
Replicate 2	Ldha -ve	28.95	29.77	Ldha	30.46	29.25
Replicate 1	Hhex -ve	29.89	30.80	Hhex	29.67	28.39
Replicate 2	Hhex -ve	29.01	29.36	Hhex	29.87	28.40
Replicate 1	Arip3a -ve	30.61	34.93	Arip3a	29.08	28.25
Replicate 2	Arip3a -ve	30.06	31.17	Arip3a	29.46	28.46
Replicate 1	Bcl6b -ve	32.96	34.26	Bcl6b	30.56	28.55
Replicate 2	Bcl6b -ve	32.13	31.68	Bcl6b	29.86	28.56
Replicate 1	Cbx3 -ve	30.45	30.33	Cbx3	30.30	30.43
Replicate 2	Cbx3 -ve	28.86	29.09	Cbx3	31.31	30.25
Replicate 1	Xrcc -ve	31.16	30.30	Xrcc	30.18	28.55
Replicate 2	Xrcc -ve	29.51	30.72	Xrcc	29.08	27.99
Replicate 1	Bcl11a1-ve	27.90	28.19	Bcl11a1	29.04	27.31
Replicate 2	Bcl11a1-ve	28.25	28.29	Bcl11a1	29.03	26.93
Replicate 1	Mki67 -ve	27.45	28.11	Mki67	29.29	26.71
Replicate 2	Mki67 -ve	27.23	28.51	Mki67	29.47	26.51

Experiment 2

	Target Name	Input 18h-neg	Rel 18h-neg	Target Name	Input-target	Rel 18h-target
Replicate 1	Ppp1ca -ve	26.99	27.68	Ppp1ca	28.36	28.02
Replicate 2	Ppp1ca -ve	27.00	26.65	Ppp1ca	28.50	28.73
Replicate 1	Nfkbie -ve	26.41	26.73	Nfkbie	27.97	25.85
Replicate 2	Nfkbie -ve	26.50	26.55	Nfkbie	27.34	25.73
Replicate 1	Ldha -ve	26.95	27.92	Ldha	27.80	27.06
Replicate 2	Ldha -ve	27.14	27.22	Ldha	27.90	27.14
Replicate 1	Hhex -ve	27.87	26.19	Hhex	27.87	26.71
Replicate 2	Hhex -ve	27.52	26.22	Hhex	27.91	26.72
Replicate 1	Arip3a -ve	28.71	28.37	Arip3a	27.68	25.98
Replicate 2	Arip3a -ve	28.96	27.88	Arip3a	27.92	26.58
Replicate 1	Bcl6b -ve	32.69	31.45	Bcl6b	28.74	26.75
Replicate 2	Bcl6b -ve	30.64	29.07	Bcl6b	28.00	26.86
Replicate 1	Cbx3 -ve	27.27	28.34	Cbx3	29.64	28.98
Replicate 2	Cbx3 -ve	27.27	27.26	Cbx3	30.41	28.91
Replicate 1	Xrcc -ve	28.45	28.40	Xrcc	27.88	26.18
Replicate 2	Xrcc -ve	27.53	27.20	Xrcc	27.50	26.26
Replicate 1	Bcl11a1-ve	28.12	28.48	Bcl11a1	28.93	27.61
Replicate 2	Bcl11a1-ve	28.19	28.37	Bcl11a1	28.81	27.70
Replicate 1	Mki67 -ve	27.94	28.15	Mki67	29.92	27.61
Replicate 2	Mki67 -ve	27.72	28.52	Mki67	29.41	27.42
