# Supplementary material for: NF-κB subunits direct kinetically distinct transcriptional cascades in antigen receptor-activated B cells
Source: Nat Immunol. 2023 Jul 31;24(9):1552–64. doi: 10.1038/s41590-023-01561-7 (PMC10457194; doi:10.1038/s41590-023-01561-7)
Supplement: Supplementary file 10 — Supporting data for Supplementary Fig. 5e. Gene expression analysis of NF-κB target genes in follicular and MZ B cells (by qRT–PCR, CT values provided). [file 41590_2023_1561_MOESM10_ESM.pdf]

Fig S5e: Gene expression analysis of NF-κB target genes in FO and MZ B cells in

Experiment 1

	Target Name	Rel KO - FO 0h	Rel KO - FO 1h	Rel KO - FO 4h	Rel KO - FO 18h
Replicate 1	Gapdh	21.46	21.34	20.94	19.57
Replicate 2	Gapdh	21.67	21.42	20.64	19.51
Replicate 1	Nfkbia	22.22	21.43	22.27	22.71
Replicate 2	Nfkbia	22.01	21.32	22.14	22.84
Replicate 1	Tnfaip3	24.31	23.77	25.32	25.89
Replicate 2	Tnfaip3	24.25	23.89	25.90	26.06
Replicate 1	Gadd45b	24.36	22.57	25.24	25.84
Replicate 2	Gadd45b	24.33	22.58	25.31	25.92
Replicate 1	Bcl2l1	29.52	29.92	28.99	26.61
Replicate 2	Bcl2l1	29.52	29.74	28.11	26.66

	Target Name	WT - FO 0h	WT - FO 1h	WT - FO 4h	WT - FO 18h
Replicate 1	Gapdh	21.65	21.95	21.26	18.27
Replicate 2	Gapdh	21.79	22.32	20.53	17.90
Replicate 1	Nfkbia	22.32	21.16	21.90	21.04
Replicate 2	Nfkbia	22.67	21.20	21.80	20.94
Replicate 1	Tnfaip3	25.35	23.47	24.04	23.37
Replicate 2	Tnfaip3	25.31	23.47	24.49	23.14
Replicate 1	Gadd45b	25.48	22.97	23.97	23.92
Replicate 2	Gadd45b	25.43	22.81	23.92	24.18
Replicate 1	Bcl2l1	29.31	27.07	24.16	22.36
Replicate 2	Bcl2l1	29.35	26.95	24.07	22.28

	Target Name	RelA cKO - FO 0h	RelA cKO - FO 1h	RelA cKO - FO 4h	RelA cKO - FO 18h
Replicate 1	Gapdh	21.31	21.95	20.69	19.69
Replicate 2	Gapdh	21.24	21.73	21.03	19.05
Replicate 1	Nfkbia	22.22	22.18	22.56	22.76
Replicate 2	Nfkbia	22.13	22.27	22.70	22.66
Replicate 1	Tnfaip3	26.30	26.95	23.87	25.29
Replicate 2	Tnfaip3	26.38	27.14	23.68	25.53
Replicate 1	Gadd45b	25.41	23.66	24.07	25.17
Replicate 2	Gadd45b	25.51	23.55	24.12	24.83
Replicate 1	Bcl2l1	29.72	28.46	24.66	23.46
Replicate 2	Bcl2l1	29.34	28.10	24.40	23.52

	Target Name	RelA fl/fl - FO 0h	RelA fl/fl - FO 1h	RelA fl/fl - FO 4h	RelA fl/fl - FO 18h
Replicate 1	Gapdh	20.52	21.40	20.57	19.75

Replicate 2	Gapdh	20.67	21.76	19.69	19.55
Replicate 1	Nfkbia	21.50	20.13	21.28	22.50
Replicate 2	Nfkbia	21.59	20.00	21.03	22.69
Replicate 1	Tnfaip3	25.87	22.39	23.05	25.20
Replicate 2	Tnfaip3	25.84	22.46	23.18	25.22
Replicate 1	Gadd45b	24.18	21.40	22.82	25.28
Replicate 2	Gadd45b	24.18	21.39	22.96	25.06
Replicate 1	Bcl2l1	27.97	24.71	23.52	23.51
Replicate 2	Bcl2l1	27.85	24.88	23.45	23.91

Experiment 2

	Target Name	Rel KO - FO 0h	Rel KO - FO 1h	Rel KO - FO 4h	Rel KO - FO 18h
Replicate 1	Gapdh	21.16	20.69	19.81	18.91
Replicate 2	Gapdh	21.28	20.90	19.51	18.93
Replicate 1	Nfkbia	20.68	19.28	20.76	22.12
Replicate 2	Nfkbia	20.60	20.41	20.78	22.14
Replicate 1	Tnfaip3	23.81	23.19	24.03	25.64
Replicate 2	Tnfaip3	23.66	22.95	24.25	25.81
Replicate 1	Gadd45b	23.09	22.58	23.40	25.59
Replicate 2	Gadd45b	23.25	22.28	23.49	25.23
Replicate 1	Bcl2l1	28.28	28.80	28.14	25.86
Replicate 2	Bcl2l1	27.81	28.42	28.16	25.72

	Target Name	WT - FO 0h	WT - FO 1h	WT - FO 4h	WT - FO 18h
Replicate 1	Gapdh	22.17	22.42	20.81	17.57
Replicate 2	Gapdh	22.14	22.45	20.96	17.57
Replicate 1	Nfkbia	22.06	21.23	22.18	20.23
Replicate 2	Nfkbia	22.55	21.05	22.11	20.11
Replicate 1	Tnfaip3	24.65	24.39	23.35	22.33
Replicate 2	Tnfaip3	24.80	23.52	23.05	21.80
Replicate 1	Gadd45b	25.08	23.25	23.87	23.45
Replicate 2	Gadd45b	25.10	23.18	23.86	23.56
Replicate 1	Bcl2l1	28.75	27.64	24.00	21.58
Replicate 2	Bcl2l1	29.08	26.42	23.72	21.66

	Target Name	RelA cKO - FO 0h	RelA cKO - FO 1h	RelA cKO - FO 4h	RelA cKO - FO 18h
Replicate 1	Gapdh	21.61	20.73	19.77	15.88
Replicate 2	Gapdh	21.74	20.78	19.74	16.06
Replicate 1	Nfkbia	22.96	21.92	21.12	19.11
Replicate 2	Nfkbia	22.81	21.26	21.11	19.17
Replicate 1	Tnfaip3	26.73	24.47	23.25	21.78
Replicate 2	Tnfaip3	26.85	24.74	23.33	21.96

Replicate 1	Gadd45b	26.32	23.76	22.78	21.48
Replicate 2	Gadd45b	25.88	23.59	22.96	22.14
Replicate 1	Bcl2l1	29.15	28.18	22.40	20.63
Replicate 2	Bcl2l1	29.24	27.94	22.84	20.56

	**Target Name**	**RelA fl/fl - FO 0h**	**RelA fl/fl - FO 1h**	**RelA fl/fl - FO 4h**	**RelA fl/fl - FO 18h**
Replicate 1	Gapdh	21.18	20.87	19.24	15.72
Replicate 2	Gapdh	20.97	20.82	19.21	15.52
Replicate 1	Nfkbia	21.94	20.20	20.29	18.43
Replicate 2	Nfkbia	21.79	20.35	20.22	18.43
Replicate 1	Tnfaip3	25.90	23.32	22.11	21.46
Replicate 2	Tnfaip3	25.45	23.34	22.05	21.22
Replicate 1	Gadd45b	25.08	22.63	22.96	22.34
Replicate 2	Gadd45b	25.29	23.07	22.89	21.72
Replicate 1	Bcl2l1	26.83	25.82	22.51	19.86
Replicate 2	Bcl2l1	26.55	25.99	22.11	19.56

ı RelA cKO and Rel KO mice

Rel KO - MZ 0h	Rel KO - MZ 1h	Rel KO - MZ 4h	Rel KO - MZ 18h
21.30	21.41	21.50	23.43
20.76	21.95	20.91	22.87
22.81	22.39	23.23	25.98
22.67	22.38	23.48	25.76
25.79	25.84	25.73	29.46
25.69	25.85	26.72	29.02
25.29	24.42	26.03	30.46
25.31	24.51	25.97	29.45
29.95	30.84	29.71	30.49
29.40	30.52	29.50	30.63

WT - MZ 0h	WT - MZ 1h	WT - MZ 4h	WT - MZ 18h
22.06	22.67	25.95	20.34
21.95	22.71	25.81	19.53
23.37	22.99	27.89	23.25
23.13	23.23	27.74	23.39
26.05	25.81	29.92	25.76
26.40	25.93	29.18	26.24
26.28	24.75	30.17	26.12
25.65	24.94	30.23	26.21
29.50	29.00	30.50	24.72
29.34	28.86	30.74	24.68

RelA cKO - MZ 0h	RelA cKO - MZ 1h	RelA cKO - MZ 4h	RelA cKO - MZ 18h
22.49	23.43	23.22	28.92
22.34	22.80	22.97	27.73
24.83	25.35	25.55	31.71
24.95	25.04	25.92	29.75
28.61	28.50	28.21	32.86
28.71	28.20	27.93	32.36
26.30	26.17	27.28	32.85
26.37	26.23	27.04	33.18
30.94	30.63	28.36	31.39
30.29	31.20	27.91	31.89

RelA fl/fl - MZ 0h	RelA fl/fl - MZ 1h	RelA fl/fl - MZ 4h	RelA fl/fl - MZ 18h
21.91	21.36	21.70	29.16

21.82	21.42	21.64	28.75
23.40	22.51	23.97	29.67
23.72	22.46	23.98	28.90
25.10	24.98	25.68	31.31
26.03	25.28	25.83	31.58
26.29	27.75	26.12	32.44
26.12	27.70	26.11	32.64
30.06	27.96	26.10	31.88
30.70	27.58	26.14	33.20

Rel KO - MZ 0h	Rel KO - MZ 1h	Rel KO - MZ 4h	Rel KO - MZ 18h
21.60	20.77	19.20	24.87
21.55	20.44	19.08	25.04
21.72	22.31	22.94	27.28
21.28	21.89	22.94	28.50
24.39	24.71	24.66	30.79
24.36	25.13	24.75	30.98
24.93	24.32	24.51	30.19
24.75	24.45	24.55	31.29
30.01	29.65	28.19	32.89
29.54	29.85	28.33	34.21

WT - MZ 0h	WT - MZ 1h	WT - MZ 4h	WT - MZ 18h
22.47	22.34	21.46	23.25
22.67	22.06	21.77	23.53
23.32	23.71	24.61	25.81
23.25	23.64	24.56	25.50
25.26	25.83	25.92	28.98
24.88	25.53	25.76	28.56
26.21	25.41	26.90	29.25
25.98	25.55	26.36	29.00
30.27	30.14	26.91	27.51
29.92	29.43	26.97	27.57

RelA cKO - MZ 0h	RelA cKO - MZ 1h	RelA cKO - MZ 4h	RelA cKO - MZ 18h
20.47	21.02	20.59	28.69
20.33	20.95	20.59	27.57
22.76	22.93	22.94	30.70
22.59	22.67	22.41	29.91
26.34	26.37	25.59	30.79
26.52	26.46	26.06	32.83

25.07	24.33	25.18	33.22
25.47	24.87	25.23	32.15
28.84	29.65	25.32	31.74
28.76	28.98	25.28	30.80

**RelA fl/fl - MZ 0h**	**RelA fl/fl - MZ 1h**	**RelA fl/fl - MZ 4h**	**RelA fl/fl - MZ 18h**
20.77	20.05	19.44	28.30
20.83	20.08	19.54	29.40
21.94	20.92	21.51	29.88
21.95	21.10	21.50	29.07
25.20	23.80	23.49	31.40
25.18	23.90	23.59	30.36
24.70	23.78	24.88	31.81
24.84	23.19	24.14	31.83
27.71	26.65	24.75	32.22
27.74	26.83	24.72	33.20
